# Harnessing Diversity towards the Reconstructing of Large Scale Gene Regulatory Networks

**DOI:** 10.1371/journal.pcbi.1003361

**Published:** 2013-11-21

**Authors:** Takeshi Hase, Samik Ghosh, Ryota Yamanaka, Hiroaki Kitano

**Affiliations:** 1 The Systems Biology Institute, Shirokanedai, Minato, Tokyo, Japan; 2 Laboratory of Disease Systems Modeling, Center for Integrative Medical Sciences, RIKEN, Suehiro-cho, Tsurumi-ku, Yokohama City, Kanagawa, Japan; 3 Department of Genome Science, RCAST, The University of Tokyo, Komaba, Meguro, Tokyo, Japan; 4 Sony Computer Science Laboratories, Inc., Higashigotanda, Shinagawa, Tokyo, Japan; 5 Okinawa Institute of Science and Technology, Onna, Onna-son, Kunigami, Okinawa, Japan; University of Chicago, United States of America

## Abstract

Elucidating gene regulatory network (GRN) from large scale experimental data remains a central challenge in systems biology. Recently, numerous techniques, particularly consensus driven approaches combining different algorithms, have become a potentially promising strategy to infer accurate GRNs. Here, we develop a novel consensus inference algorithm, Top*k*Net that can integrate multiple algorithms to infer GRNs. Comprehensive performance benchmarking on a cloud computing framework demonstrated that (i) a simple strategy to combine many algorithms does not always lead to performance improvement compared to the cost of consensus and (ii) Top*k*Net integrating only high-performance algorithms provide significant performance improvement compared to the best individual algorithms and community prediction. These results suggest that a priori determination of high-performance algorithms is a key to reconstruct an unknown regulatory network. Similarity among gene-expression datasets can be useful to determine potential optimal algorithms for reconstruction of unknown regulatory networks, i.e., if expression-data associated with known regulatory network is similar to that with unknown regulatory network, optimal algorithms determined for the known regulatory network can be repurposed to infer the unknown regulatory network. Based on this observation, we developed a quantitative measure of similarity among gene-expression datasets and demonstrated that, if similarity between the two expression datasets is high, Top*k*Net integrating algorithms that are optimal for known dataset perform well on the unknown dataset. The consensus framework, Top*k*Net, together with the similarity measure proposed in this study provides a powerful strategy towards harnessing the wisdom of the crowds in reconstruction of unknown regulatory networks.

## Author Summary

Elucidating gene regulatory networks is crucial to understand disease mechanisms at the system level. A large number of algorithms have been developed to infer gene regulatory networks from gene-expression datasets. If you remember the success of IBM's Watson in ”Jeopardy!„ quiz show, the critical features of Watson were the use of very large numbers of heterogeneous algorithms generating various hypotheses and to select one of which as the answer. We took similar approach, “Top*k*Net”, to see if “Wisdom of Crowd” approach can be applied for network reconstruction. We discovered that “Wisdom of Crowd” is a powerful approach where integration of optimal algorithms for a given dataset can achieve better results than the best individual algorithm. However, such an analysis begs the question “How to choose optimal algorithms for a given dataset?” We found that similarity among gene-expression datasets is a key to select optimal algorithms, i.e., if dataset A for which optimal algorithms are known is similar to dataset B, the optimal algorithms for dataset A may be also optimal for dataset B. Thus, our “Top*k*Net” together with similarity measure among datasets can provide a powerful strategy towards harnessing “Wisdom of Crowd” in high-quality reconstruction of gene regulatory networks.

## Introduction

Most genes do not exert their functions in isolation [1], but make their functions through regulations among them. Such regulatory interactions are in the same cell, between different cells, and even between different organs, forming large-scale gene regulatory networks (GRNs). The impact of genetic abnormality can spread through regulatory interactions in GRNs and alter the activity of other genes that do not have any genetic defects [2]. Analyses of GRNs are key to identify disease mechanisms and possible therapeutic targets for the future [1]. Therefore, reconstruction of accurate and comprehensive GRNs from genome-wide experimental data (*e.g.*, gene expression data from DNA microarray experiments) is one of the fundamental challenges in systems biology [3], [4].

A plethora of algorithms have been developed to infer GRNs from gene expression data, *i.e.*, mutual-information (MI) based algorithms [5]–[12], correlation-based algorithms [5], Bayesian networks (BNs) [13]–[17], regression-based algorithms [18]–[22], graphical gaussian model (ggm) [23], meta predictors that combine several different methods [24], [25], and several other approaches that were recently proposed [26]–[32], *i.e.*, random forests based algorithm [26] (GENIE3) and two-way ANOVA based algorithm [27] (ANOVA). Each network-inference algorithm generates a confidence score for a link between two genes from expression data and assumes that a predicted link with higher confidence score is more reliable. Systematic and comparative assessment of the performance of these inference algorithms remains a major challenge in network reconstruction [33].

Several studies compared performances of the network-inference algorithms [7], [8], [9], [34]. Especially, the DREAM5 (Dialogue on Reverse Engineering Assessment and Methods) challenge evaluated performances of many and diverse network-inference algorithms (29 algorithms submitted by challenge participants and 6 commonly used “off-the-shelf” algorithms) by using benchmark dataset composed of large-scale *Escherichia coli*, *Saccharomyces cerevisiae*, and in silico regulatory networks and their corresponding expression datasets [35]. The evaluation demonstrated that no single individual algorithm performs optimally across all the three expression-datasets, *i.e.*, GENIE3 and ANOVA perform optimally for *E. coli* dataset, while two algorithms based on regression techniques are optimal for in silico dataset. Further, algorithm-specific biases influence the recovery of different regulation patterns, *i.e.*, MI and correlation based algorithms can recover feed-forward loops most reliably, while regression and BNs can more accurately recover linear cascades than MI and correlation based algorithms [35].

Above observations suggest that different network-inference algorithms have different strengths and weaknesses [33], . A natural corollary to the observations is that combining multiple network-inference algorithms may be a good strategy to infer an accurate and comprehensive GRN. Recently, Marbach et al. proposed a new network-inference algorithm, “Community Prediction”, by combining several network-inference algorithms that were submitted to DREAM5 challenge [35]. The Community Prediction combining 29 algorithms (“off-the-self” algorithms are not used) shows higher or at least comparable performance to the best among the 29 algorithms across all DREAM5 datasets. Further, performance of community prediction increases as the number of integrated algorithms increases. Thus, community prediction based on integration of many algorithms can be a robust approach to infer GRNs across diverse datasets and will provide a powerful framework to reconstruct unknown regulatory networks.

Analysis of DREAM5 results [35] reveal that algorithms complement each other in a context-specific manner and harnessing the combined strengths and weaknesses of diverse techniques can lead to high quality inference networks. Thus, it is important to analyze the anatomy of diversity and quantify it. This is particularly important to systematically evaluate the characteristics of individual techniques and leverage their diversity in finding an optimal combination set for a specific experimental data context. Recently, Marbach et al. showed that integration of algorithms with high-diversity outperform that with low-diversity [35]. However, their diversity analysis is qualitative and, to our knowledge, there is no measure to quantify algorithm diversity.

Analysis of small in silico datasets of the DREAM3 challenge demonstrated that integration of the best five algorithms outperforms integration of all algorithms submitted to the challenge [33]. Selection of optimal algorithms for a given expression data and integration of the selected optimal algorithms may be more powerful strategy to reconstruct accurate GRNs than using many algorithms. Development of a method to determine optimal algorithms is a key to reconstruct accurate and comprehensive GRNs, although it is difficult to identify beforehand optimal algorithms for reconstruction of an unknown regulatory network because of biological and experimental variations among expression datasets.

A measure to quantify similarity among gene-expression datasets could be a clue to determine the optimal algorithms for reconstruction of unknown regulatory networks. This is because, if expression-data associated with known regulatory network (e.g., the DREAM5 datasets) is similar to that with unknown regulatory network, optimal algorithms for data with known regulatory network could be also optimal for data with unknown regulatory network.

Motivated by the above observations and issues, this paper focuses on four strategies towards building a comprehensive network reconstruction platform –

- A computational framework to integrate diverse inference algorithms.
- Systematically assess the performance of the framework against the DREAM5 datasets composed of genome-wide transcriptional regulatory networks and their corresponding expression data from actual microarray experiments as well as in silico simulation.
- Develop a measure to quantify diversity among inference techniques towards identifying optimal combination of algorithms which elucidate accurate GRNs.
- Develop a measure to quantify similarity among expression datasets towards selecting optimal algorithms for reconstruction of unknown regulatory networks.

To investigate these possible strategies, we first develop a novel network-inference algorithm that can combine multiple network-inference algorithms. Second, to evaluate inference performances of the algorithms precisely, we used the DREAM5 datasets composed of *E. coli* and *S. cerevisiae* transcriptional regulatory networks and their corresponding expression data from large-scale microarray experiments, together with synthetic network and corresponding expression datasets (http://wiki.c2b2.columbia.edu/dream/index.php/D5c4). A cloud-based computing framework was developed on the Amazon Web Services (AWS) system to systematically benchmark the large data-sets and compute-intensive algorithms. Third, we define a mathematical function quantifying diversity between algorithm pairs to analyze the anatomy of diversity and its role in improving the performance of reverse engineering techniques. Finally, we present a similarity measure among expression-datasets and its potential to identify optimal algorithms for reconstruction of unknown regulatory networks.

## Results

**Figure 1:**
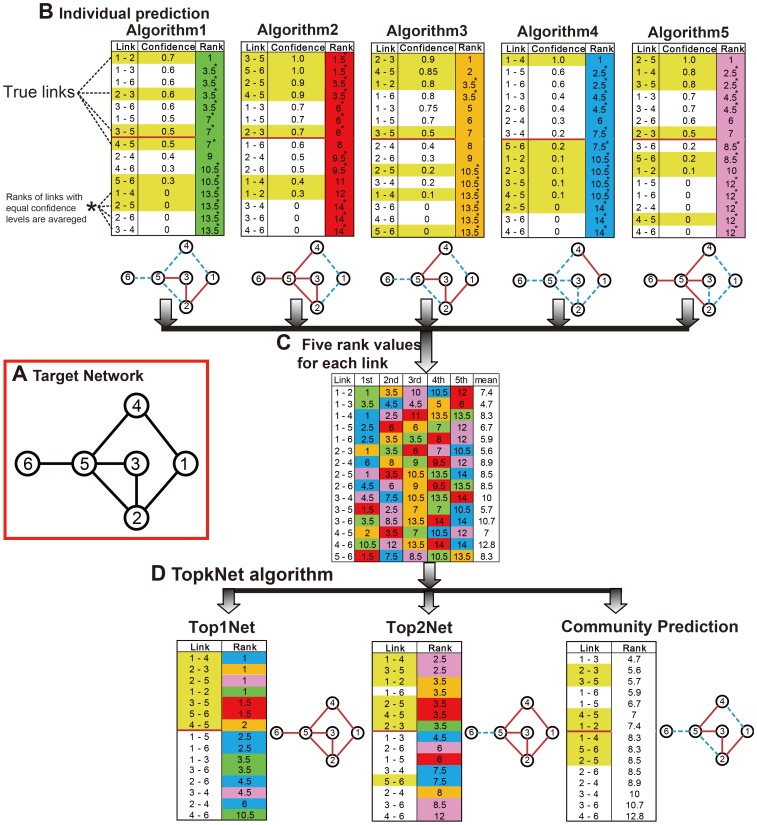
Example of a prediction by Top*k*Net formed from five individual network predictions. (**a**) Target Network. Circles and links are genes and regulatory links among genes, respectively. (**b**) The five lists are ranked according to the confidence levels of links, the most reliable prediction is at the top of the list and has the highest rank, *i.e.*, Algorithm1 assigns the highest confidence level and the rank value of 1 to a link between nodes 1 and 2. The true link of the target network is highlighted in yellow. We regard links with rank of 1–7 as regulatory links inferred by the algorithms because the target network composed of 7 links. Red lines and blue dashed lines represent true positive and false negative links, respectively. (**c**) Five rank values for each link and the mean value among the five values. Green, red, orange, blue, and purple represent rank values from Algorithm1, Algorithm2, Algorithm3, Algorithm4, and Algorithm5, respectively. (**d**) Rank value of a link by Top*k*Net and that by Community Prediction. Top1Net and Top2Net regards 1st and 2nd highest value among five rank values for a link as the rank value of the link, respectively. Community Prediction calculates the mean value among five rank values for a link and regards the mean as the rank of the link. For example, rank of the links between genes 1 and 2 for Community Prediction is 7.4. This example illustrates how Top1Net can be more accurate than the other algorithms.

We developed a computational workflow for the combination of network-inference algorithms and systematic assessment of their performance. The workflow of our framework is composed of three steps (see Supplementary figure S1 and Materials and Methods for details):

- **Inference Methods**: We obtained confidence score between gene pairs based on 29 algorithms submitted by DREAM5 participants and 6 commonly used “off the shelf” algorithms from Marbach et al [35]. Furthermore, we calculated confidence score between two genes based on other three algorithms, *i.e.*, c3net [9], ggm [23], and mrnet [8] algorithms. We used, in total, 38 network-inference algorithms for the study.
- **Top** ***k*** **Net**: A novel algorithm to generate predicted list of regulatory links by the network-inference algorithms that can combine multiple network-inference algorithms (in this case, 38 algorithms) (see Figure 1).
- **Performance Assessment**: Comparative evaluation of the performance of Top*k*Net with that of the 38 network-inference algorithms and Community Prediction, benchmarked using the DREAM5 network-inference challenge dataset composed of the large synthetic data (number of genes = 1,643 and sample size = 805), large-scale *E. coli* and *S. celevisiae* networks (number of gene = 4,511 and 5,950, respectively), and their corresponding real microarray gene expression data (with sample size of 805 and 536, respectively). Table 1 summarizes the different data-sets employed in the performance assessment for this study. We used a cloud-computing infrastructure built on Amazon EC2 instances to infer GRNs from the large-scale DREAM5 dataset (see Materials and Methods for details).

**Table 1:** The DREAM5 datasets used in this study.

-	In silico^1^	*E. coli* 2	*S. celevisiae* 3
Number of genes	1,643	4,511	5,950
Number of samples	805	805	536

1 In silico Dream 5 dataset.

2 Dream 5 dataset from *E.coli*.

3 Dream5 dataset from *S.cerevisiae*.

**Top** *k* **Net** (the maximum value of *k* is the number of integrated algorithms, 38 in this case) is based on leveraging the diversity of the different techniques by combining the confidence of each gene pair interaction computed by the algorithms. Top*k*Net applied a bagging method, which was introduced by Breiman L [36], to combine confidence scores between each gene pair from multiple network inference algorithms. Top1Net assumes that two genes have a regulatory links between them, if at least one network-inference algorithm assigns high confidence level to the link between them, while Top38Net assumes that two genes have a regulatory link between them, only if all the 38 algorithms assign high confidence levels to the link between them (see below and Figure 1 for details). Top*k*Net with *k* = 2–37 and Community prediction (which takes the average of ranks assigned by different algorithms), are intermediates between Top1Net and Top38Net.

**Figure** 1 gives an illustrative example of **Top** *k* **Net** (for simplicity, in this illustrative example, we used 5 individual algorithms) on a sample target network (Figure 1A). As shown in Figure 1B, a network-inference algorithm assigns a confidence level to each link and links are ranked according to their confidence levels, *i.e.*, a link with higher confidence level has higher rank value. For each link, 5 individual network-inference algorithms (represented by different colors) assigned 5 rank values to each link (Figure 1C). Among the five rank values of each link, **Top** *k* **Net** regards *k*th highest rank value as the rank value of the link (Figure 1D). For example, five rank values (1, 3.5, 10, 10.5, and 12) are assigned to the regulatory link between nodes 1 and 2 (Figure 1C). In this case, **Top**1**Net** and **Top**2**Net** regards 1 and 3.5 as the rank value of the link, respectively (see Figure 1D). As shown in color spectrums in Figure 1D, **Top** *k* **Net** algorithms reconstruct GRNs which include predicted connections from multiple algorithms.

Based on the observation that network-inference algorithms tend to assign high confidence levels to true-positive links [6], [34], **Top**1**Net** algorithm would infer the largest number of true-positive links among all algorithms, *i.e.*, **Top** *k* **Net** algorithms would infer smaller number of true-positive links as the value of *k* increases and, at the same time, can avoid inferring false-positive links. Thus, in general, **Top**1**Net** would outperform other algorithms in terms of inference performances, as seen in the predicted network in Figure 1.

### Comparative performance assessment

Network inference algorithms have increased following Moore's Law (doubling every two years) [33], [37]. Consequently, it has become increasing important to develop comprehensive performance benchmarking platforms to compare their relative strengths and weaknesses and leverage them to improve quality of inferred network. Two key components fundamental to performance assessment are representative metrics to quantify performance and standardized data sets on which to evaluate them.

In this section, we first outline these components employed in this study on the basis of which the performance of Top*k*Net is evaluated.

#### Benchmarking data sets

The performance of gene network reconstruction algorithms require benchmarking against various data sets representing network dynamics (for example, gene expression profiles) for which the underlying network is known. However, the ability to generate biologically plausible networks and validate them against experimental data remains a fundamental tenet in network reconstruction. In this respect, the DREAM initiative provides a community platform for the objective assessment of inference methods. The DREAM challenges provide a common framework on which to evaluate inference techniques against well-characterized data sets. In this study, we used large scale experimental data from the DREAM5 network inference challenge.

#### Performance metrics

 True-positive rate, false-positive rate, recall, and precision are representative metrics to evaluate performances of network inference algorithms (see Materials and Methods for details). True-positive (false-positive) rate is, among all true (false) links, how many of them are with ranks beyond a threshold. Precision is, among all links with ranks beyond a threshold, how many of them are true links, while recall is, among all true links, how many of them are with ranks beyond a threshold. For example, in Figure 1, Top2Net for threshold of 3.5 shows recall = 6/7 (one missing) and precision = 6/7 (one mistake). By changing threshold gradually, we obtained a receiver operating characteristic (ROC) and Precision/recall (PR) curves that are graphical plots of true-positive rate vs. false-positive rate and precision vs. recall, respectively. These curves are straightforward visual representation of performances of network-inference algorithms.

Further, we calculated three representative metrics, *i.e.*, AUC-PR (area under the PR curve), AUC-ROC (area under the ROC curve), and max f-score (f-score is harmonic mean of precision and recall) for these algorithms (see Materials and Methods for details). AUC-PR and AUC-ROC evaluate the average performances of network-inference algorithms, while max f-score evaluates the optimal performance of network-inference algorithms. A network-inference algorithm with higher inference performance would show higher AUC-PR, AUC-ROC, and max f-score. Moreover, DREAM5 also provides performance benchmarking package which computes an overall score (OS) across the entire dataset [35]. By using the package, we also calculated overall score to evaluate performance of Top*k*Net algorithms against community prediction and 38 algorithms (for performance of the individual 38 algorithms, see Supplementary table S1).

### TopkNet performance on DREAM5 data set

**Figure 2:**
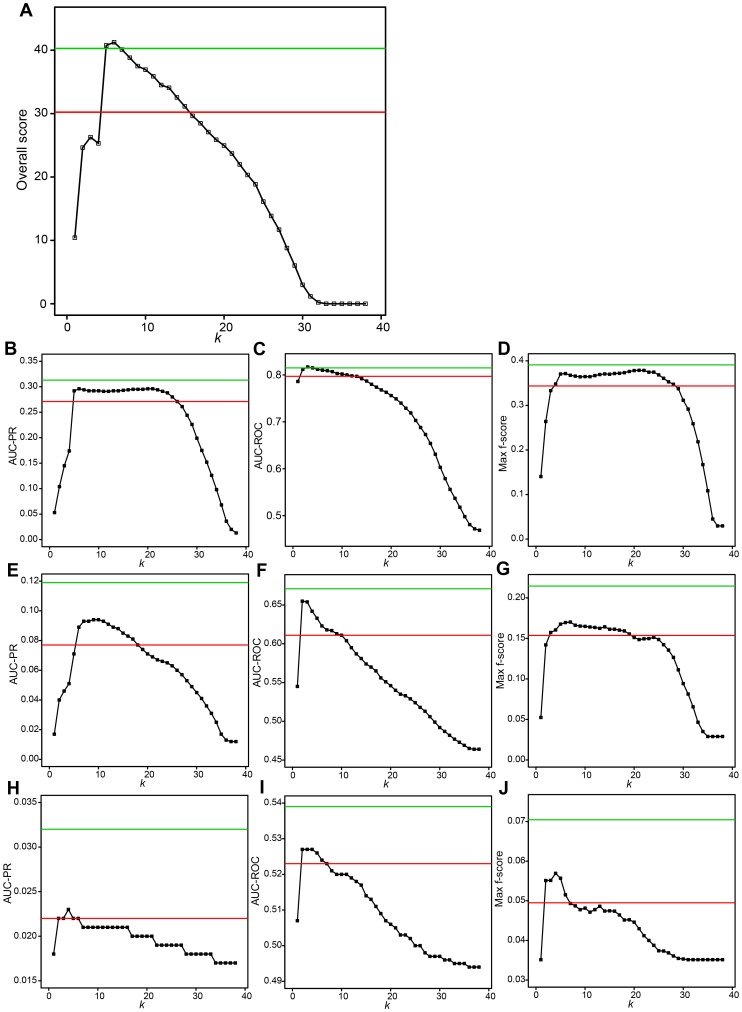
Performances of Top*k*Net and community prediction based on integration of the 38 network-inference algorithms. Black squares and lines show performances of Top*k*Net algorithm. For example, values at *k* = 1 represent performances of Top1Net algorithm. Red and green lines represent performances of community prediction and those of the best algorithm, respectively. (**A**) Overall score. (**B**) AUC-PR for in silico dataset. (**C**) AUC-ROC for in silico dataset. (**D**) Max f-score for in silico dataset. (**E**) AUC-PR for *E. coli* dataset. (**F**) AUC-ROC for *E. coli* dataset. (**G**) Max f-score for *E. coli* dataset. (**H**) AUC-PR for *S. cerevisiae* dataset. (**I**) AUC-ROC for *S. cerevisiae* dataset. (**J**) Max f-score for *S. cerevisiae* dataset.

To evaluate how Top*k*Net leverages diversity amongst the candidate algorithms to infer consensus network, we used the DREAM5 benchmarking data comprised of large scale synthetic and experimental gene regulatory networks for *E.coli* and *S.cerevesiae* as outlined in Table 1 and computed different performance metrics on them. As seen in the PR and ROC curves for in silico, *E. coli*, and *S. cerevisiae* datasets (Supplementary figures S2 and S3), Top6net shows constantly higher performance compared to community prediction. Other three performance metrics (AUC-PR, AUC-ROC, and Max f-score) of Top*k*Net with *k* = 5–8 are also higher than those of community prediction for all the three datasets (see Figures 2B–J). Thus, overall score of Top*k*Net with *k* = 5–8 is significantly higher than that of community prediction (see Figure 2B). However, the performance metrics of Top*k*Net is only comparable to the best individual algorithms and not significantly better. Community prediction also showed significantly lower performances than the best individual algorithm.

These results indicate that, while Top*k*Net would provide better strategy to integrate multiple algorithms than community prediction, such a strategy does not always significant increase in performance compared to the cost of integration. As seen in this section, the overall score of the best individual algorithm (40.279) is comparable to that of Top*k*Net with *k* = 5–7 (40.110–41.251) and is much higher than that of community prediction and TopkNet with *k* = 1 (30.228 and 10.432, respectively). This is because, for the DREAM5 datasets, several low-performance algorithms assign high confidence scores to many false-positive links and such false links could decrease the performance of Top*k*Net (especially, with *k* = 1) and community prediction algorithms.

**Figure 3:**
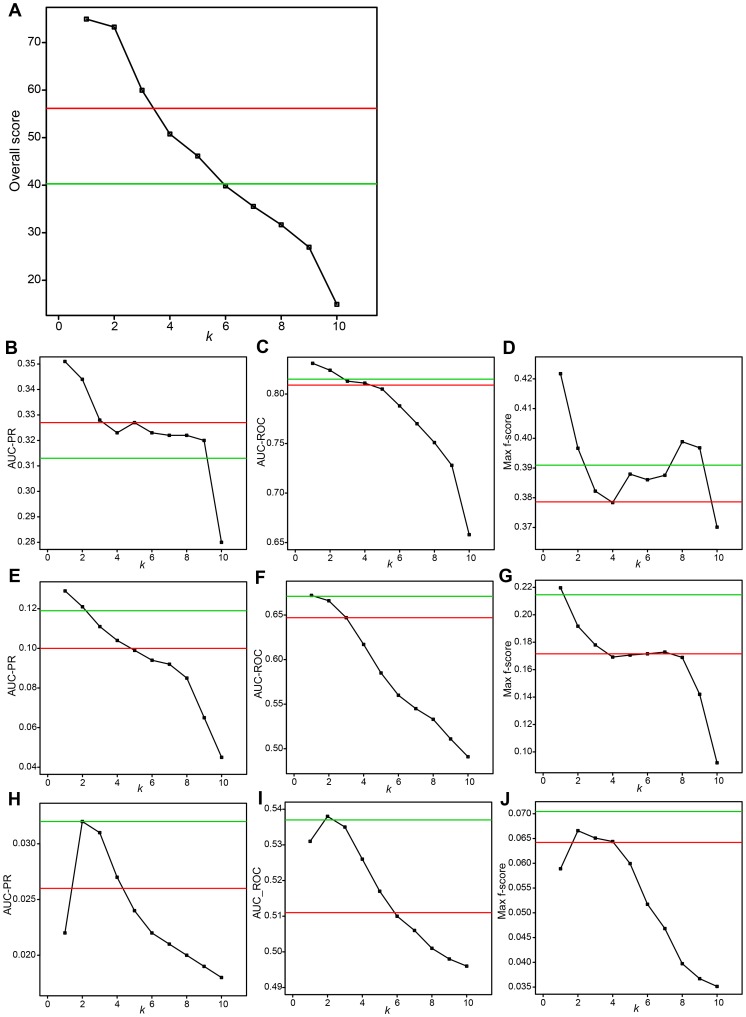
Performances of Top*k*Net and Community prediction based on integration of top 10 highest-performance algorithms. Black squares and lines show performances of Top*k*Net algorithm. For example, values at *k* = 1 represent performances of Top1Net algorithm. Red and green lines represent performances of community prediction and those of the best algorithm, respectively. (**A**) Overall score. (**B**) AUC-PR for in silico dataset. (**C**) AUC-ROC for in silico dataset. (**D**) Max f-score for in silico dataset. (**E**) AUC-PR for *E. coli* dataset. (**F**) AUC-ROC for *E. coli* dataset. (**G**) Max f-score for *E. coli* dataset. (**H**) AUC-PR for *S. cerevisiae* dataset. (**I**) AUC-ROC for *S. cerevisiae* dataset. (**J**) Max f-score for *S. cerevisiae* dataset.

Thus, by integrating only high-performance algorithms that tend to assign high confidence score to true-positive link, Top*k*Net (especially, with *k* = 1) and community prediction may show much higher performances than the best individual algorithms. To investigate this issue, we evaluate Top*k*Net (and community prediction) based on integration of 10 optimal algorithms (algorithms within top 10 highest AUC-PR) for each of the in silico, *E. coli*. and *S. cerevisiae* datasets. As seen in Supplementary figures S4 and S5, PR and ROC curves of Top1Net are constantly over those of the best individual algorithm and community prediction for in silico and *E. coli* datasets, although, for *S. cerevisiae*, PR-curve of the best individual algorithm slightly over that of Top1Net. Other three metrics (AUC-PR, AUC-ROC, and Max f-score) of Top*k*Net with low *k* (*k* = 1 for in silico and *E. coli* and *k* = 2 for *S. cerevisiae*) are significantly higher or at least comparable to those of the best individual algorithm and community prediction (see Figures 3B–J). Therefore, the overall score of Top*k*Net with *k* = 1 and 2 (74.935 and 73.261, respectively) are significantly higher than that of the best individual algorithm (40.279) and community prediction (56.158) (see Figure 3A). These results highlight that integration of multiple high-performance algorithms by Top1Net or Top2Net consistently reconstructs the most accurate GRNs for different datasets.

As demonstrated in this section, selection of optimal algorithms for a given expression data and Top1Net, Top2Net, and community prediction based on integration of the selected optimal algorithms could be a powerful approach to reconstruct high-quality GRNs. However, currently, to our knowledge, there is no method to determine beforehand optimal algorithms for expression data associated with an unknown regulatory network. Development of a method to determine optimal algorithms is a key to reconstruct unknown regulatory networks (We investigate this issue in the next section).

### Selection of optimal algorithm pairs to infer GRNs based on algorithm diversity

Different network-inference algorithms employ different and often complementary techniques to infer gene regulatory interactions from an expression dataset. Therefore, a consensus driven approach, which leverages *diversity* in network-inference algorithms, can infer more accurate and comprehensive GRNs than a single network-inference algorithm. However, as demonstrated in this study, a simple strategy of increasing the number of algorithms may not always yield significant performance gains compared to the *cost of consensus*, *i.e.*, the computation cost (CPU time and memory usage).

It is pertinent to analyze the anatomy of diversity between different algorithms in a theoretical framework to answer the questions of -

- To what extent, then, are the algorithms different from each other?
- Does bringing diversity of the algorithms into community prediction improve the quality of inferred networks?

For the purposes, Marbach et al. conducted principal component analysis (PCA) on confidence scores from 35 network-inference algorithms [35]. They mapped 35 algorithms onto 2^nd^ and 3^rd^ principal components and grouped the algorithms into four clusters by visual inspection. The analysis demonstrated that integration of three algorithms from different clusters shows higher performance than that from the same cluster. It indicates that the diversity signature of the selected algorithms, and not just the number of algorithms, plays an important role in the performance of the network reconstruction techniques.

**Figure 4:**
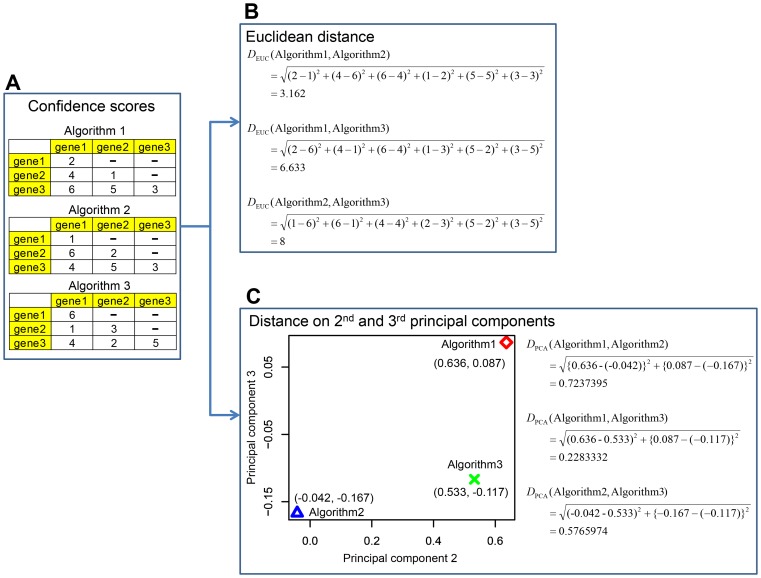
A toy example to calculate diversity among algorithms. (**A**) Confidence scores from algorithms. Confidence score of a link between two genes were generated by each of three algorithms. In this case, each algorithm has 6 confidence scores for 6 links. Note that the three algorithms in this example are algorithms to infer non-directional algorithms and make symmetrical matrices of confidence scores, i.e., confidence score of link from gene1 to gene 2 is same as that from gene2 to gene1. Thus, for simplicity, upper triangles of confidence score matrices are not shown in the figure. (**B**) Diversity among algorithms based on Euclidean distances. In this example, each of three algorithms has a vector of 6 confidence scores for 6 links between two genes. Euclidean distance between two vectors of confidence scores from two algorithms is calculated and is defined as diversity between the two algorithms. (**C**) Diversity among algorithms based on 2nd and 3rd components of PCA analysis. In this example, PCA analysis is conducted on three vectors of 6 confidence scores from three network-inference algorithms and the three algorithms are mapped on to 2nd and 3rd principal components (see left panel of C). Euclidean distance between two algorithms is calculated by using the 2nd and 3rd principal components and is defined as diversity between the two algorithms.

However, their algorithm diversity is qualitative and, to our knowledge, there is no quantitative measure for algorithm diversity. In order to quantify diversity among the individual algorithms employed in this study, we developed two quantitative measures of diversity which calculates distance between algorithms pairs on the basis of confidence scores of regulatory interactions inferred by the algorithms. One is based on simple Euclidean distance (EUC distance) and the other is based on EUC distance on 2^nd^ and 3^rd^ components from PCA analysis (PCA distance) (see Materials and Methods for details). In Figure 4, we provide a toy model to explain how diversity among network-inference algorithms is defined.

By using the diversity measures, we calculated distance among 10 optimal algorithms for each of the DREAM5 datasets to examine whether bringing quantified algorithm diversity into Top1Net (and Community prediction) improves the performances of network reconstruction. Based on the calculated distances, we defined high-diversity pairs as top 10% of algorithm pairs with highest distance, while low-diversity pairs are defined as bottom 10% of algorithm pairs with lowest distance. In this study, we have 45 algorithm pairs among 10 optimal algorithms and thus top 5 algorithm pairs with highest distance are high-diversity pairs, while bottom 5 algorithm pairs with lowest distance are low-diversity pairs.

**Figure 5:**
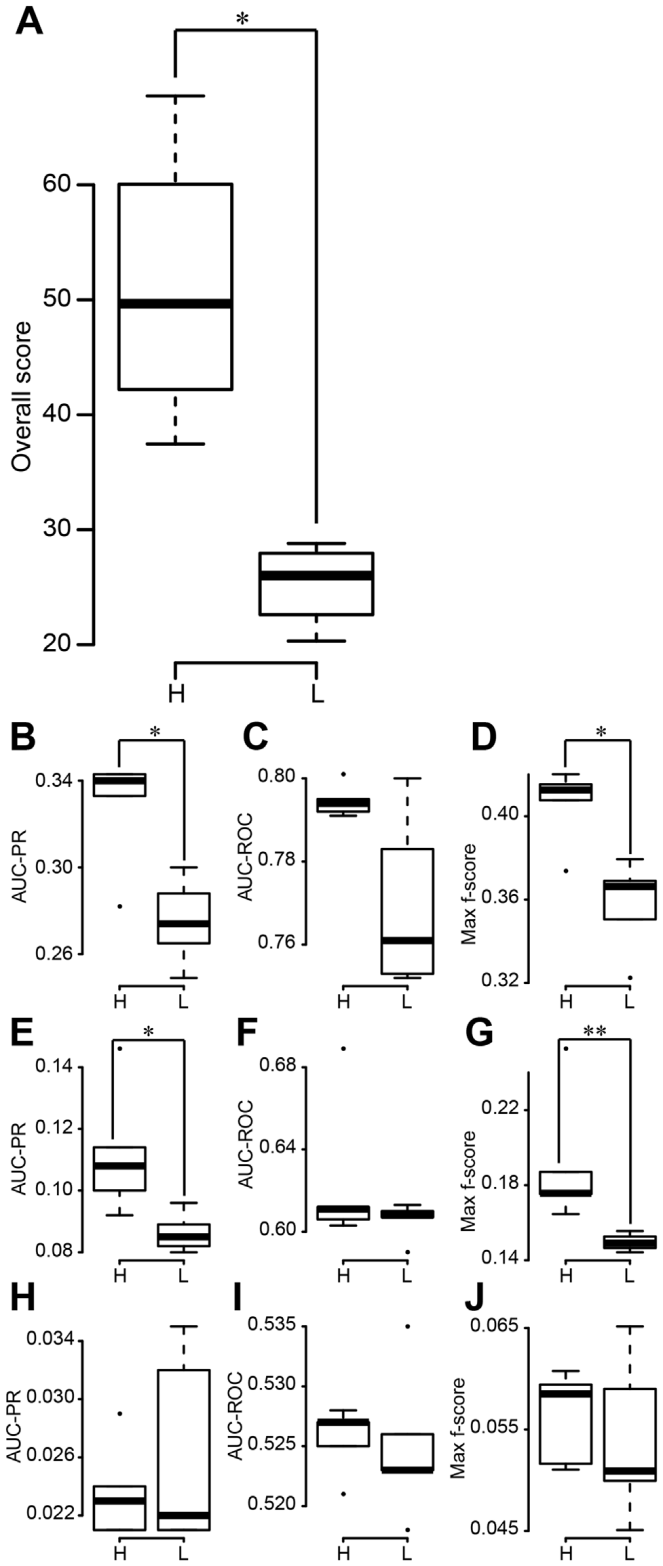
Performances of Top1Net based on integration of high- or low-diversity algorithm pairs by EUC distance. H and L represent high-diversity and low-diversity algorithm pairs, respectively. (**A**) Box-plots of overall score. (**B**) Box-plots of AUC-PR for in silico dataset. (**C**) Box-plots of AUC-ROC for in silico dataset. (**D**) Box-plots of Max f-score for in silico dataset. (**E**) Box-plots of AUC-PR for *E. coli* dataset. (**F**) Box-plots of AUC-ROC for *E. coli* dataset. (**G**) Box-plots of Max f-score for *E. coli* dataset. (**H**) Box-plots of AUC-PR for *S. cerevisiae* dataset. (**I**) Box-plots of AUC-ROC for *S. cerevisiae* dataset. (**J**) Box-plots of max f-score for *S. cerevisiae* dataset. * and ** represent P<0.05 and P<0.01, by the Wilcoxon rank sum test.

Next, we evaluated the performances of Top1Net (or community prediction) based on integration of high-diversity pairs and those of low-diversity pair. As seen in Figures 5B–J and Supplementary figures S6B–J, AUC-PR, AUC-ROC, and max f-score of high-diversity pairs by EUC distance are higher or at least comparable to those of low-diversity pairs by EUC distance across all datasets. Especially, for *in silico* and *E. coli* datasets, AUC-PR and Max f-score of high-diversity pairs by EUC distance are significantly higher than that of low-diversity pairs by EUC distance. Thus, the overall score of high-diversity pairs is also significantly higher than that of low-diversity pair (P<0.05) (see Figure 5A and Supplementary figure S6A). The performances of high-diversity pairs by PCA distance are also higher or at least comparable to those of low-diversity pairs by PCA distance (see Supplementary figures S7 and S8). Furthermore, median value of the overall score of high-diversity pairs (47.725 and 50.250 by Top1Net, for EUC and PCA distances, respectively) are much higher than that by the best individual algorithms (40.279) and that by community prediction that integrates 38 network-inference algorithms (30.228). In summary, these results indicate that -,

- Even for the same number of algorithms (in this case, two algorithms are integrated), the quantitative diversity to selected pairs can improve the performance of the consensus methods (Top*k*Net and community prediction).
- Quantitative diversity-guided consensus can reduce the cost of consensus (only 2 algorithms integration instead of 38 algorithms integration in this case) without compromising the quality of the inferred network as shown in this study where the inference performance of high diversity pair is much higher than that of 38 algorithms combination.

### Selection of optimal algorithms based on similarity among expression datasets towards reliable reconstruction of regulatory networks

Top1Net or Top2Net based on integration of highest-performance algorithms consistently reconstruct the most accurate GRNs, as demonstrated in the previous section (see Figure 3). However, as Marbach et al. mentioned, “*Given the biological variation among organisms and the experimental variation among gene-expression datasets, it is difficult to determine beforehand which methods will perform optimally for reconstruction an unknown regulatory network*” [35], and, to our knowledge, there is no method to select the optimal network-inference algorithms. Development of a method to select optimal network-inference algorithms for each of the expression datasets remains a major challenge in network reconstruction.

A measure to quantify similarity among expression datasets can be a key to select optimal network-inference algorithms for each of the datasets, because, if similarity between expression-data associated with known regulatory network (*e.g.*, DREAM5 datasets) and that with unknown regulatory network is high, optimal algorithms for the known dataset can be repurposed to infer regulatory network from unknown dataset. Driven by this observation, we developed a similarity measure among gene-expression datasets based on algorithm diversity proposed in previous section.

**Figure 6:**
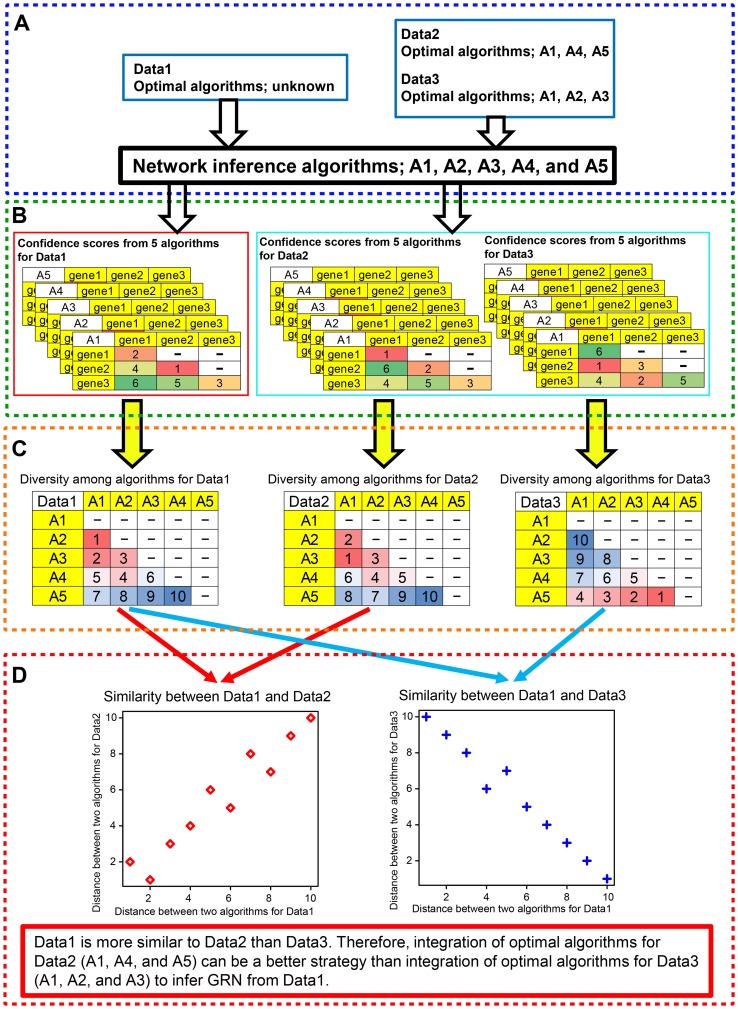
Overview of a method to calculate similarity between two expression datasets. (**A**) Datasets. Expression datasets were split into a dataset for which optimal algorithms are unknown (e.g., Data1) and datasets for which optimal algorithms are known (e.g., Data2 and Data3). (**B**) Confidence scores of links between two genes. For each of datasets, confidence scores from each of algorithms (e.g., algorithms, A1, A2, A3, A4, and A5) were calculated. (**C**) Diversity among algorithms. By using confidence scores calculated in (B), diversity among algorithms were calculated for each of three datasets. In this example, we examined five algorithms and thus, for each of the datasets, we have a vector of 10 distances between two algorithms. (**D**) Similarity between two expression datasets. Correlation coefficient between the vector of algorithm distances from Data1 and that from Data2 was calculated. The calculated correlation coefficient is defined as similarity between Data1 and Data2. In the example in this figure, Data1 is more similar to Data2 than Data3. Thus, optimal algorithms for Data2 could perform better than those for Data3 to infer GRN from Data1.

First, we briefly explain the overview of the procedure to calculate similarity among expression datasets (see Figure 6 and Materials and Methods for the details). The procedure is composed of 4 steps. (1) The expression datasets were split into a dataset for which optimal algorithms are unknown (e.g, Data1 in Figure 6A) and datasets for which optimal algorithms are known (e.g., Data2 and Data3 in Figure 6A). (2) For each of the datasets, confidence scores of links were calculated by network-inference algorithms. In the example shown in Figure 6B, each of 5 algorithms calculates 6 confidence scores for 6 links. (3) By using the confidence scores calculated in the step (2), diversity among algorithms was calculated based on a distance measure proposed in the previous section (EUC and PCA based distances, see Figure 6C and Materials and Methods for details), for each of the datasets. In the example shown in Figure 6C, we have 10 algorithm pairs among 5 algorithms and thus, as shown in matrices in the figure, we have 10 distances between two algorithms for each of the three datasets. (4) By using algorithm diversity calculated in the step (3), we calculated correlation coefficient of the algorithm distances between two datasets (see Figure 6D). In terms of algorithm diversity, the correlation coefficient is regarded as similarity measure between the two datasets. In the example shown in Figure 6D, Data1 is more similar to Data2 than Data3. Thus, optimal algorithms for Data2 are better fit than those for Data3 to infer GRN from Data1.

**Figure 7:**
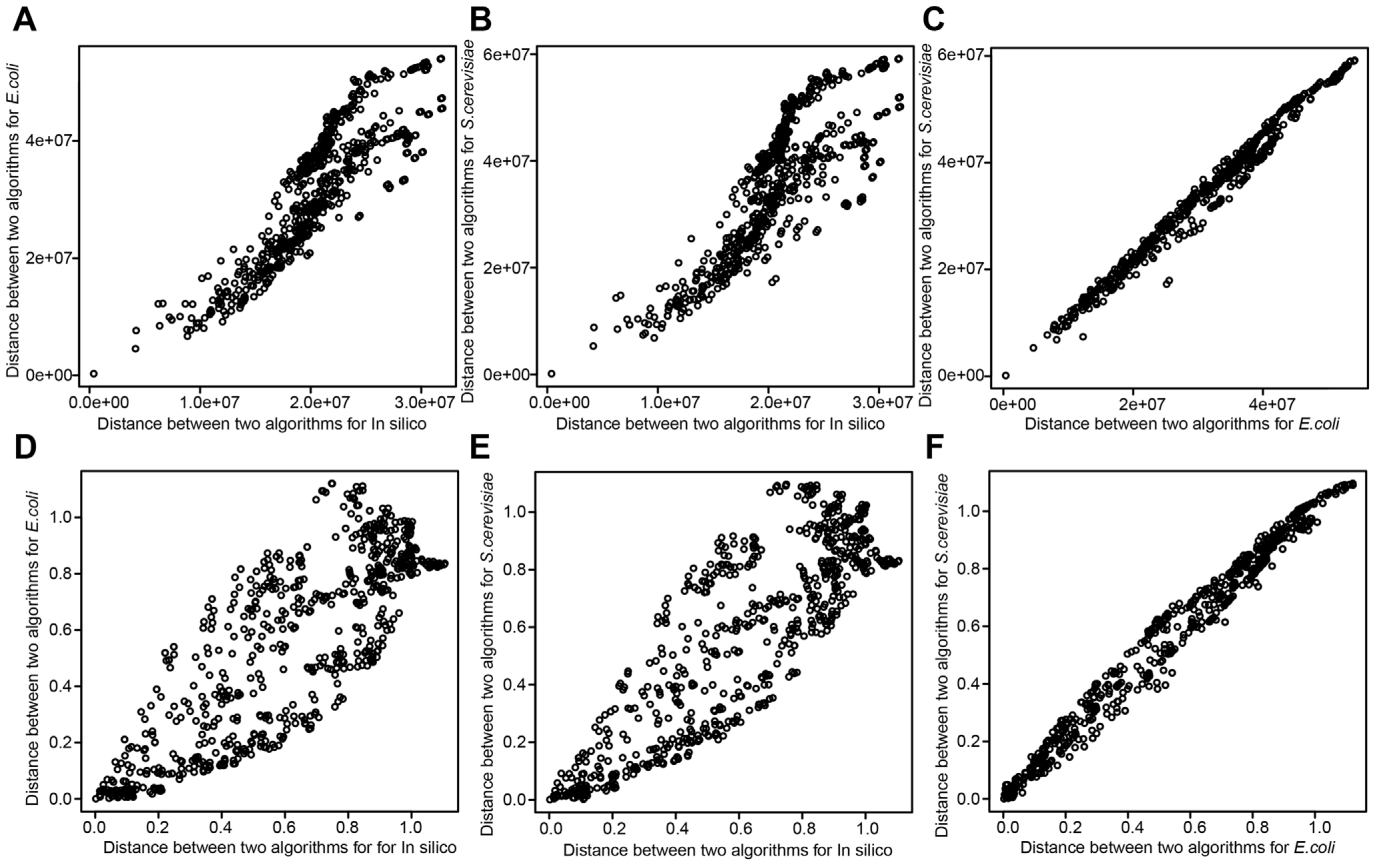
Similarity among gene-expression datasets based on algorithm diversity. The scatter plots show correlation of algorithm distance between two gene-expression datasets. Each of points in scatter plots represents each of algorithm pairs. Because we have 703 algorithm pairs among 38 algorithms, 703 points are in each of the figures. Vertical axis represents (EUC or PCA) distance between two algorithms for one gene-expression dataset, while horizontal axis represents that for the other gene-expression dataset. (**A**) Scatter plots of EUC distance for in silico and *E. coli* datasets. (**B**) Scatter plots of EUC distance for in silico and *S. cerevisiae* dataset. (**C**) Scatter plots of EUC distance for *E. coli* and *S. cerevisiae* datasets (**D**) Scatter plots of PCA distance for in silico and *E. coli* datasets. (**E**) Scatter plots of PCA distance for in silico and *S. cerevisiae* datasets. (**F**) Scatter plots of PCA distance for *E. coli* and *S. cerevisiae* datasets.

Next, to evaluate whether dataset similarity can be used to govern optimal selection of inference algorithms, we calculated the similarity among the DREAM5 gene-expression datasets and compared the performance of the algorithms across the datasets. As seen in Figure 7 and Table 2, correlation between *S. cerevisiae* and *E. coli* datasets (Spearman's correlation coefficient *ρ* = 0.99) is much higher than that between *E. coli* and *in silico* (*ρ* = 0.87 and 0.81 by EUC and PCA distances, respectively) and that between *S. cerevisiae* and *in silico* (*ρ* = 0.83). In terms of algorithm diversity, similarity between *E. coli* and *S. cerevisiae* datasets is much higher than that between *E. coli* and *in silico* and that between *S. cerevisiae* and *in silico*.

**Table 2:** Correlation coefficient of algorithm distances and that of performance metrics across the DREAM5 gene-expression datasets.

Dataset 1	Dataset 2	EUC distance^1^	PCA distance^2^
**In silico** 3	***E.coli*** 4	0.87	0.81
**In silico**	***S.cerevisiae*** 5	0.83	0.83
***E.coli***	***S.cerevisiae***	0.99	0.99

1 Spearman's correlation coefficient of algorithm distance (EUC distance) between Dataset 1 and Dataset 2.

2 Spearman's correlation coefficient of algorithm distance (PCA distance) between Dataset 1 and Dataset 2.

3 In silico Dream 5 dataset.

4 Dream 5 dataset from *E.coli*.

5 Dream5 dataset from *S.cerevisiae*.

Further correlation of algorithm performances between dataset pair with high similarity (*e.g.*, *E. coli* and *S. cerevisiae* pair) is higher than that between dataset pair with low similarity (*e.g.*, *in silico* and *E. coli* pair and *in silico* and *S. cerevisiae* pair) (see Supplementary figure S7 and Supplementary Table S2). These results indicate that, for dataset pair with high similarity, optimal network-inference algorithms for one dataset also tend to be optimal for the other dataset.

From above observations (observations in Figure 7, Supplementary figure S9, Table 2, and Supplementary table S2), we hypothesized that, if similarity between the two expression-datasets is high, integration of algorithms that are optimal for one dataset could perform well on the other dataset. To examine this issue in more detail, we integrated algorithms that are optimal for *S. cerevisia* dataset (algorithms with 10 highest AUC-PR values on the dataset) and those for the in silico dataset and evaluated their performance of these two integrations against *E. coli* dataset.

**Figure 8:**
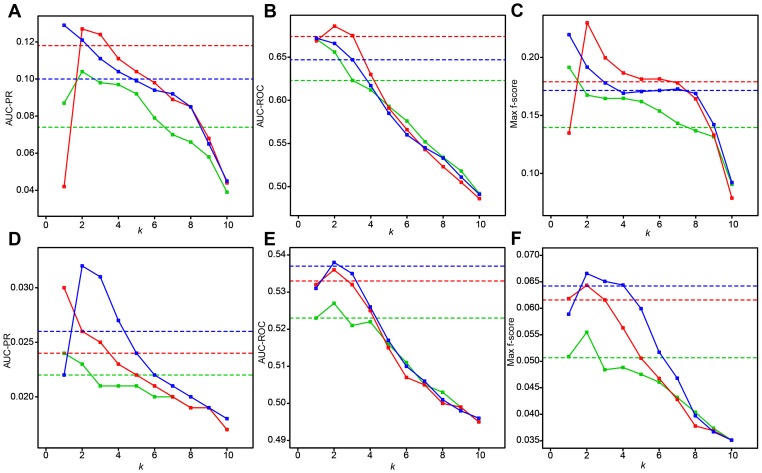
Optimal algorithm selection based on similarity among expression datasets and its potential to improve network-inference accuracy. Red lines show performance of Top*k*Net integrating algorithms that are optimal for a dataset with high-similarity, while green lines show that with low-similarity. Blue lines show performance of Top*k*Net integrating top 10 highest-performance algorithms. Dashed lines in red, green, and blue represent performance of community prediction integrating algorithms that are optimal for a dataset with high-similarity, that with low-similarity, and top 10 highest-performance algorithms, respectively. (**A**) AUC-PR for *E. coli* dataset. (**B**) AUC-ROC for *E. coli* dataset. (**C**) Max f-score for *E. coli* dataset. (**D**) AUC-PR for *S. cerevisiae* dataset. (**E**) AUC-ROC for *S. cerevisiae* dataset. (**F**) Max f-score for *S. cerevisiae* dataset.

As seen in Figures 8A, B, and C, against the *E.coli* dataset, performances (AUC-PR, AUC-ROC, and max f-score) of optimal integration from *S. cerevisiae* dataset (green lines) are generally higher than those from in silico dataset (red lines). Further, against the *S. cerevisiae* dataset, we evaluate performances of optimal-algorithm integration from *E. coli* dataset and that for in silico dataset and found that optimal integration from *E. coli* dataset (green lines) generally outperform that from in silico dataset (red lines) (see Figures 8D, E, and F). Because similarity between *S. cerevisiae* and *E. coli* datasets are much higher than that between *E. coli* and in silico datasets and that between *S. cerevisiae* and in silico datasets (see Figure 7 and Table 2), these results support the above hypothesis.

 Further, as shown in Figure 8, performance of Top*k*net integrating optimal algorithms from a dataset with high-similarity (green lines) is comparable to that integrating top 10 highest-performance algorithms (blue lines). Thus, data-similarity based optimal algorithm selection together with Top*k*Net (or community prediction) based integration of the selected optimal algorithms can be a powerful strategy to reconstruct unknown regulatory network.

## Discussion

With an increasing corpus of inference algorithms, leveraging their diverse and sometimes complementary approaches in a community consensus can be a promising strategy for reconstruction of gene regulatory networks from large scale experimental data. A computational platform to systematically analyze, assess and leverage these diverse techniques is essential for the successful application of reverse engineering in biomedical research.

This study presents a reverse engineering framework which can flexibly integrate multiple inference algorithms, based on **Top** *k* **Net** - a novel technique for building a consensus network based on the algorithms. It is pertinent to note here that the consensus framework based on **Top** *k* **Net** can be flexibly extended to include various types of network-inference algorithms.

Comparative evaluation on the DREAM5 datasets showed that, although Top*k*Net based on 38-algorithm integration shows lower or at most comparable performance to the best individual algorithms, Top1Net based on integration of top 10 highest performance algorithms significantly outperforms the best individual algorithm as well as community prediction. The results demonstrated that (i) a simple strategy to combine many algorithms does not always lead to performance improvement compared to the cost of consensus and (ii) selection of high-performance algorithms for a given expression dataset and Top1Net based on integration of the selected high-performance algorithms could be a powerful strategy for reliable reverse engineering.

Why does Top1net algorithm integrating 10 optimal algorithms perform quite well and outperform the best individual method? This is because 10 optimal algorithms tend to assign high-confidence scores to true-positive links and Top1net method can recover many true-positive links that are with the highest confidence scores from 10 optimal algorithms. Furthermore, 10 optimal algorithms are based on different techniques (*e.g.*, mutual information, regression, and other statistical techniques) and Top1net can leverage diversity from the optimal algorithms. For example, the optimal algorithms based on mutual-information and regression techniques can accurately recover true positive links in feed-forward loops and linear cascade modules, respectively [35], while Top1net could integrate the algorithms and accurately recover both feed-forward loops and linear cascade module in a GRN. Therefore, Top1net shows higher inference performance than the best individual algorithms.

Why, then, Top1net outperforms community prediction and Topknet with higher *k*? Community prediction and Topknet with larger *k* recover links with lower confidence scores than Top1net, *i.e.*, community prediction uses mean among confidence scores from 10 optimal algorithms and Topknet uses *k*th highest confidence score from the algorithms. Links with lower confidence scores from optimal algorithms are more likely to be false-positive links and thus Top1net shows higher inference performance than community prediction and Topknet with higher *k*.

A key to reconstruct accurate GRNs is development of a method to determine optimal algorithms for a given expression dataset associated with unknown regulatory network. As mentioned in results, if similarity between expression-data associated with known regulatory network (*i.e.*, DREAM5 datasets) and that with an unknown regulatory network is high, optimal algorithms for data with known regulatory network may be also optimal for reconstruction of the unknown regulatory network.

Based on this observation, we developed a measure to quantify similarity among the expression datasets based on algorithm diversity and demonstrated that, if similarity between the two expression-datasets is high, integration of algorithms that are optimal for one dataset could perform well on the other dataset. Thus, the similarity measure proposed in this study can be a good clue to identify optimal algorithms for reliable reconstruction of an unknown regulatory network.

 The consensus framework outlined in this paper, Top*k*Net, together with analysis of similarity among expression datasets, provide a powerful platform towards harnessing the *wisdom of the crowds* approach in reconstruction of large scale gene regulatory networks.

## Materials and Methods

### DREAM5 datasets

We used the DREAM5 datasets (http://wiki.c2b2.columbia.edu/dream/index.php/D5c4) to evaluate performance of network-inference algorithms. The DREAM5 dataset composed of an in-silico network (1,643 genes), the real transcriptional regulatory network of *E. coli* (4,511 genes), that of *S. celecisiae* (5,950 genes), and corresponding expression dataset (805, 805, and 536 samples for the in-silico, *E. coli*, and *S. celevisiae* networks, respectively). The expression dataset of *E. coli* and that of *S. celevisae* are composed of hundreds of experiments, *i.e.*, genetic, drug, and environmental perturbations. The in-silico network is generated by extracting a subnetwork composed of 1,643 genes from the *E. coli* transcriptional network. The expression datasets of the in-silico network was simulated by software GeneNetWeaver version 2.0 [38]. For the DREAM5 datasets, in the same manner to Marbach et al. [35], we used the links with the top 100,000 highest confidence scores by each network-inference algorithm to evaluate performance of the algorithm.

To evaluate performance of inference algorithms for the DREAM5 datasets, DREAM organizers provide a matlab software (http://wiki.c2b2.columbia.edu/dream/index.php/D5c4). The software calculates 4 metrics for each network, *i.e.*, AUC-PR, AUC-ROC, AUC-PR p-value, and AUC-ROC p-value. AUC-PR (AUC-ROC) p-value is the probability that a given or greater AUC-PR (AUC-ROC) is obtained by random scoring of links. Furthermore, the software calculates an overall score that was used to evaluate the overall performance of the algorithms for all three networks (the large synthetic network, large real *E. coli*, and *S. celevisiae* GRNs) of the DREAM5 network inference challenge. The overall score (OS) is defines as OS = 0.5(*p*_1_+*p*_2_), where *p*_1_ and *p*_2_ are the mean of the log-transformed AUC-PR p-values and that of the log-transformed AUC-ROC p-values taken over the three networks of the DREAM5 challenge, respectively.

### Confidence score of regulatory links from 38 network-inference algorithms

We obtained confidence scores between two genes by 35 algorithms (29 algorithms are from DREAM5 participants and 6 algorithms are commonly used “off-the shelf” algorithms) from supplementary file of Marbach et al. [35]. For c3net, ggm, and mrnet algorithms, we calculated confidence scores of regulatory link by using GeneNet package [39], c3net R package [9], [10], and minet R package [40], respectively. Because Marbach et al. used links with top 100,000 highest confidence scores from each of 35 algorithms for analyses [35], we used top 100,000 links from c3net, ggm, and mrnet for analyses in this study.

### Metrics to evaluate performance of inference algorithms

For a given threshold value of confidence level, network-inference algorithms predict whether a pair of genes have regulatory link or not. A pair of genes with a predicted link is considered as a true positive (TP) if the link is present in the underlying synthetic network, while the pair is a false positive (FP) if the synthetic network does not have the link. Similarly, a pair of genes without a predicted link is considered as a true negative (TN) or false negative (FN) depending on whether the link exists or not in the underlying synthetic network, respectively. By using the values of TP, FP, TN, and FN, we can calculate several metrics to evaluate performances of network-inference algorithms.

One representative metric is precision/recall curve where the precision (*p*) and recall (*r*) are defined as 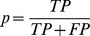 and 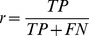, respectively. By using many threshold values, we obtained a precision/recall curve that is a graphical plot of the precision vs. the recall and is a straight forward visual representation of performances of network-inference algorithms. The area under the precision/recall curve (AUC-PR) is a summary metric of precision/recall curve and measures the average accuracy of network-inference algorithms. Another representative metric is ROC curve that is a graphical plot of the true-positive rate vs. the false-positive rate. The area under the ROC curve (AUC-ROC) also represents the average inference performance of algorithms. On the other hand, max f-score [41] evaluates optimum performance of network-inference algorithms where f-score is defined as harmonic mean of the precision and recall (
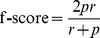
). As predictions of network-inference algorithms become more accurate, the value of AUC-PR, AUC-ROC, and max f-score becomes higher. We used AUC-PR, AUC-ROC, and max f-score for performance evaluation. To obtain these three metrics, we used package provided by the DREAM5 team [35] (PR curve, ROC curve, AUC-PR, AUC-ROC, and overall score) and perl script provided by Küffner et al. (max f-score) [27].

### Distances among network-inference algorithms

By using confidence scores among genes by network-inference algorithms, we calculated, *D*_EUC_(X,Y), the simple Euclidean distance between two network-inference algorithms (EUC distance) X and Y for expression datasets with given number of genes and given sample size. Before giving a definition for *D*_EUC_(X,Y), let us first define some notations. Let *n* be number of genes in the expression dataset and *CS*(*i*, *j*, X) be confidence value between genes *i* and *j* by algorithm X on the expression dataset. G = {(1,2), (2,3), … (*i*,*j*) …(*n*-1,*n*)} represents the list of all possible combinations of two genes for *n* genes. We defined the EUC distance between the two algorithms as 
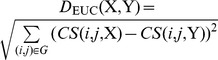
.

 Further, we calculated, *D*_PCA_(X,Y), the distance between two network-inference (X and Y) on 2^nd^ and 3^rd^ principal components (PCA distance) from PCA analysis on confidence scores of 38 algorithms. Let *PC*_2_(X) and *PC*_3_(X) be the 2^nd^ and 3^rd^ components of X, respectively. We defined the PCA distance between two algorithms as 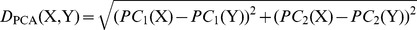. For the PCA analysis, we used R code, pricomp2.R, obtained from http://aoki2.si.gunma-u.ac.jp/R/src/princomp2.R.

### Similarity between two expression datasets based on algorithm diversity

By using distances among algorithms, we calculated, *S*(da1,da2), similarity between two expression datasets da1 and da2. Before giving definition of *S*(da1,da2), let us first define some notation. Let *k* and A = {a_1_, a_2_, …, a*_i_*, …, a*_k_*} be the number of algorithms and the list of the algorithms, respectively. AC = {(a_1_,a_2_),(a_2_,a_3_),…,(a*_i_*_-1_,a*_i_*),…, (a*_k_*_-1_,a*_k_*)} represents all possible combinations of two algorithms among *k* algorithms (*k*(*k*-1)/2 algorithm combinations). For example, in this study, we examined 38 algorithms and have 38*37/2 = 703 algorithm combinations. *D*(a*_i_*,a*_j_*,da1) represents distances between two algorithms a_1_ and a_2_ for da1. **D**_da1_{AC} = {*D*(a_1_,a_2_,da1), *D*(a_2_,a_3_,da1), …, *D*(a*_i_*_-1_,a*_i_*,da1), … *D*(a*_k_*_-1_,a*_k_*,da1)} represents a vector of *k*(*k*-1)/2 algorithm distances for da1 (in this study, we have a vector of 703 algorithm distances for each of DREAM5 datasets). We defined *S*(da1,da2) as Spearman's correlation coefficient between two vectors, **D**_da1_{AC} and **D**_da2_{AC}.

### Cloud computing infrastructure on Amazon EC2 to infer GRNs from the large-scale DREAM5 expression datasets

To infer GRNs from the large-scale expression data of DREAM5 (expression data of *E.coli* and *S. cerevisiae*), we built a cloud computing infrastructure on Amazon EC2 “High-memory double” instances (34.2 GB memory and 4 virtual cores with 3.25 EC2 Compute Units each) with Redhad linux and R version 2.15.0 [42]. We placed all the input data on the ephemeral storage disk (850 GB) of the Amazon EC2 instances and Top*k*Net output results (*e.g.*, a listing of confidence scores between genes) to files on the storage disk.

## Supporting Information

Figure S1 **The work flow of the experimental framework of this study.** Expression datasets were obtained from the DREAM5 challenge web page (http://wiki.c2b2.columbia.edu/dream/index.php/The_DREAM_Project). Inferred network from the expression datasets by a network-inference algorithm is compared to the networks of the DREAM5 challenge (Step (iii)). See Materials and Methods for details.
(TIF)

Figure S2 **PR curves of Top** ***k*** **Net and community prediction based on integration of the 38 individual algorithms.** (**A**) PR curves for in silico datasets. (**B**) PR curves for *E. coli* dataset. (**C**) PR curves for *S. cerevisiae* dataset. Vertical and horizontal axes represent precision and recall, respectively.
(TIF)

Figure S3 **ROC curves of Top** ***k*** **Net and community prediction based on integration of the 38 individual algorithms.** (**A**) ROC curves for in silico datasets. (**B**) ROC curves for *E. coli* dataset. (**C**) ROC curves for *S. cerevisiae* dataset. Vertical and horizontal axes represent true-positive and false-positive rate, respectively.
(TIF)

Figure S4 **PR curves of Top** ***k*** **Net and community prediction based on integration of the top 10 highest-performance algorithms.** (**A**) PR curves for in silico datasets. (**B**) PR curves for *E. coli* dataset. (**C**) PR curves for *S. cerevisiae* dataset. Vertical and horizontal axes represent precision and recall, respectively.
(TIF)

Figure S5 **ROC curves of Top** ***k*** **Net and community prediction based on integration of the top 10 highest-performance algorithms.** (**A**) ROC curves for in silico datasets. (**B**) ROC curves for *E. coli* dataset. (**C**) ROC curves for *S. cerevisiae* dataset. Vertical and horizontal axes represent true-positive and false-positive rate, respectively.
(TIF)

Figure S6 **Performances of community prediction based on integration of high- or low-diversity algorithm pairs by EUC distance.** H and L represent high-diversity and low-diversity algorithm pairs, respectively. (**A**) Box-plots of overall score. (**B**) Box-plots of AUC-PR for in silico dataset. (**C**) Box-plots of AUC-ROC for in silico dataset. (**D**) Box-plots of Max f-score for in silico dataset. (**E**) Box-plots of AUC-PR for *E. coli* dataset. (**F**) Box-plots of AUC-ROC for *E. coli* dataset. (**G**) Box-plots of Max f-score for *E. coli* dataset. (**H**) Box-plots of AUC-PR for *S. cerevisiae* dataset. (**I**) Box-plots of AUC-ROC for *S. cerevisiae* dataset. (**J**) Box-plots of max f-score for *S. cerevisiae* dataset. * and ** represent P<0.05 and P<0.01, by the Wilcoxon rank sum test.
(TIF)

Figure S7 **Performances of Top1Net based on integration of high- or low- diversity algorithm pairs by PCA distance.** H and L represent high-diversity and low-diversity algorithm pairs, respectively. (**A**) Box-plots of overall score. (**B**) Box-plots of AUC-PR for in silico dataset. (**C**) Box-plots of AUC-ROC for in silico dataset. (**D**) Box-plots of Max f-score for in silico dataset. (**E**) Box-plots of AUC-PR for *E. coli* dataset. (**F**) Box-plots of AUC-ROC for *E. coli* dataset. (**G**) Box-plots of Max f-score for *E. coli* dataset. (**H**) Box-plots of AUC-PR for *S. cerevisiae* dataset. (**I**) Box-plots of AUC-ROC for *S. cerevisiae* dataset. (**J**) Box-plots of max f-score for *S. cerevisiae* dataset. * represents P<0.05 by the Wilcoxon rank sum test.
(TIF)

Figure S8 **Performances of community prediction based on integration of high- or low- diversity algorithm pairs by PCA distance.** H and L represent high-diversity and low-diversity algorithm pairs, respectively. (**A**) Box-plots of overall score. (**B**) Box-plots of AUC-PR for in silico dataset. (**C**) Box-plots of AUC-ROC for in silico dataset. (**D**) Box-plots of Max f-score for in silico dataset. (**E**) Box-plots of AUC-PR for *E. coli* dataset. (**F**) Box-plots of AUC-ROC for *E. coli* dataset. (**G**) Box-plots of Max f-score for *E. coli* dataset. (**H**) Box-plots of AUC-PR for *S. cerevisiae* dataset. (**I**) Box-plots of AUC-ROC for *S. cerevisiae* dataset. (**J**) Box-plots of max f-score for *S. cerevisiae* dataset. * and ** represent P<0.05 and P<0.01, by the Wilcoxon rank sum test.
(TIF)

Figure S9 **Comparison of algorithm performances across gene-expression datasets.** The scatter plots show correlation of algorithm performance between two gene-expression datasets. Vertical axis represents algorithm performance for one gene-expression dataset, while horizontal axis represents that for the other gene-expression dataset. (**A**) Scatter plots of AUC-PR for in silico and E. coli datasets. (**B**) Scatter plots of AUC-PR for in silico and *S. cerevisiae* datasets. (**C**) Scatter plots of AUC-PR for *E. coli* and *S. cerevisiae* datasets. (**D**) Scatter plots of AUC-ROC for in silico and *E. coli* datasets. (**E**) Scatter plots of AUC-ROC for in silico and *S. cerevisiae* datasets. (**F**) Scatter plots of AUC-ROC for *E. coli* dataset and *S. cerevisiae* datasets. (**G**) Scatter plots of max f-score for in silico and *E. coli* datasets. (**H**) Scatter plots of max f-score for in silico and *S. cerevisiae* datasets. (**I**) Scatter plots of max f-score for *E. coli* and *S. cerevisiae* datasets.
(TIF)

Table S1 **Performances of the 38 individual algorithms.** The table shows overall score, AUC-PR, and AUC-ROC of the 38 algorithms.
(XLS)

Table S2 **Correlation coefficient of performance metrics across the DREAM5 gene-expression datasets.** The table shows Spearman's correlation coefficient of performance metrics across the DREAM5 gene expression datasets.
(DOC)
