# Supplementary figures and images for: Harnessing Diversity towards the Reconstructing of Large Scale Gene Regulatory Networks
Source: PLoS Comput Biol. 2013 Nov 21;9(11):e1003361. doi: 10.1371/journal.pcbi.1003361 (PMC3836705; doi:10.1371/journal.pcbi.1003361)

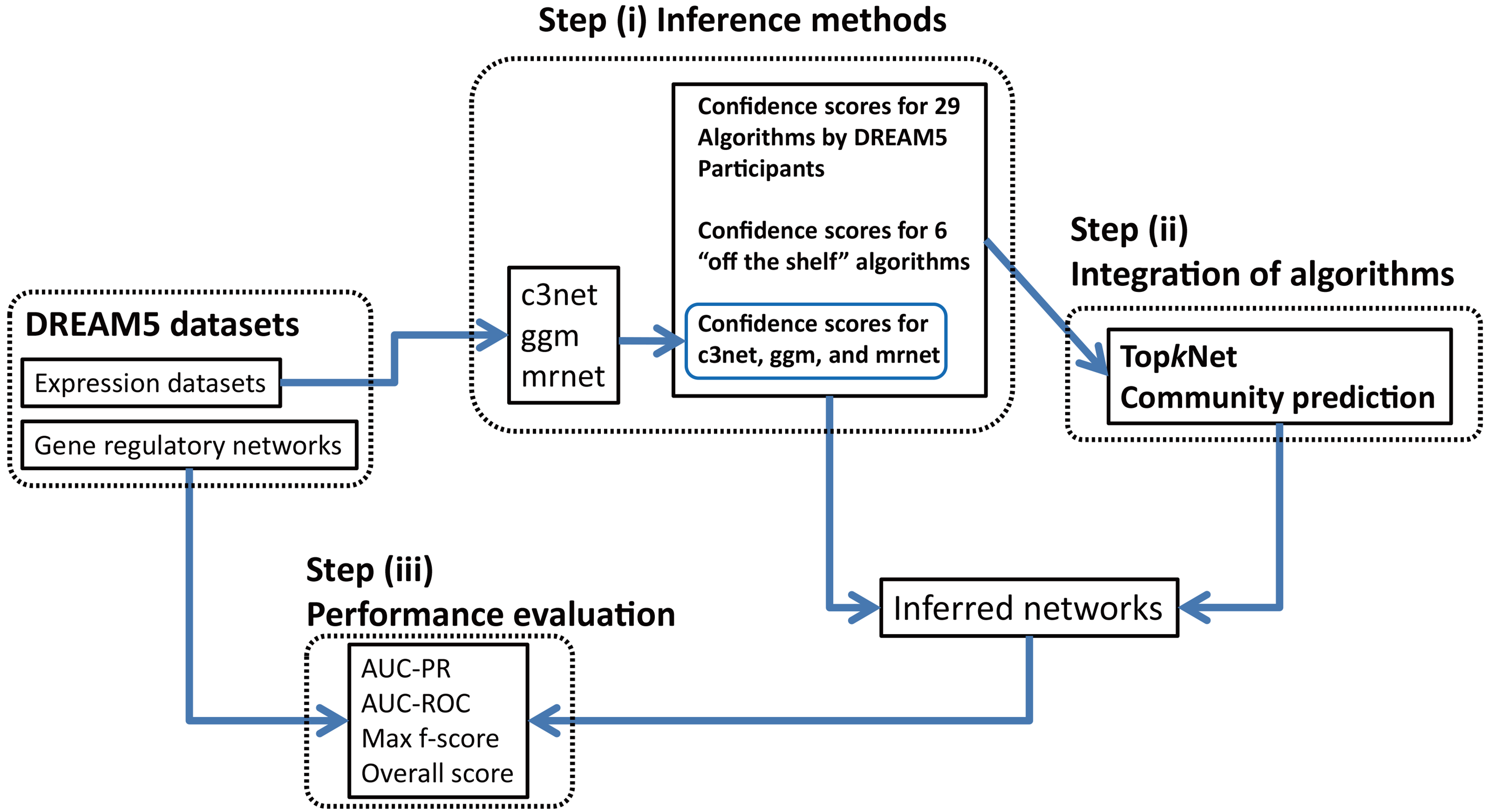

Supplement: Figure S1 — The work flow of the experimental framework of this study. Expression datasets were obtained from the DREAM5 challenge web page (http://wiki.c2b2.columbia.edu/dream/index.php/The_DREAM_Project). Inferred network from the expression datasets by a network-inference algorithm is compared to the networks of the DREAM5 challenge (Step (iii)). See Materials and Methods for details. (TIF) [file pcbi.1003361.s001.tif]

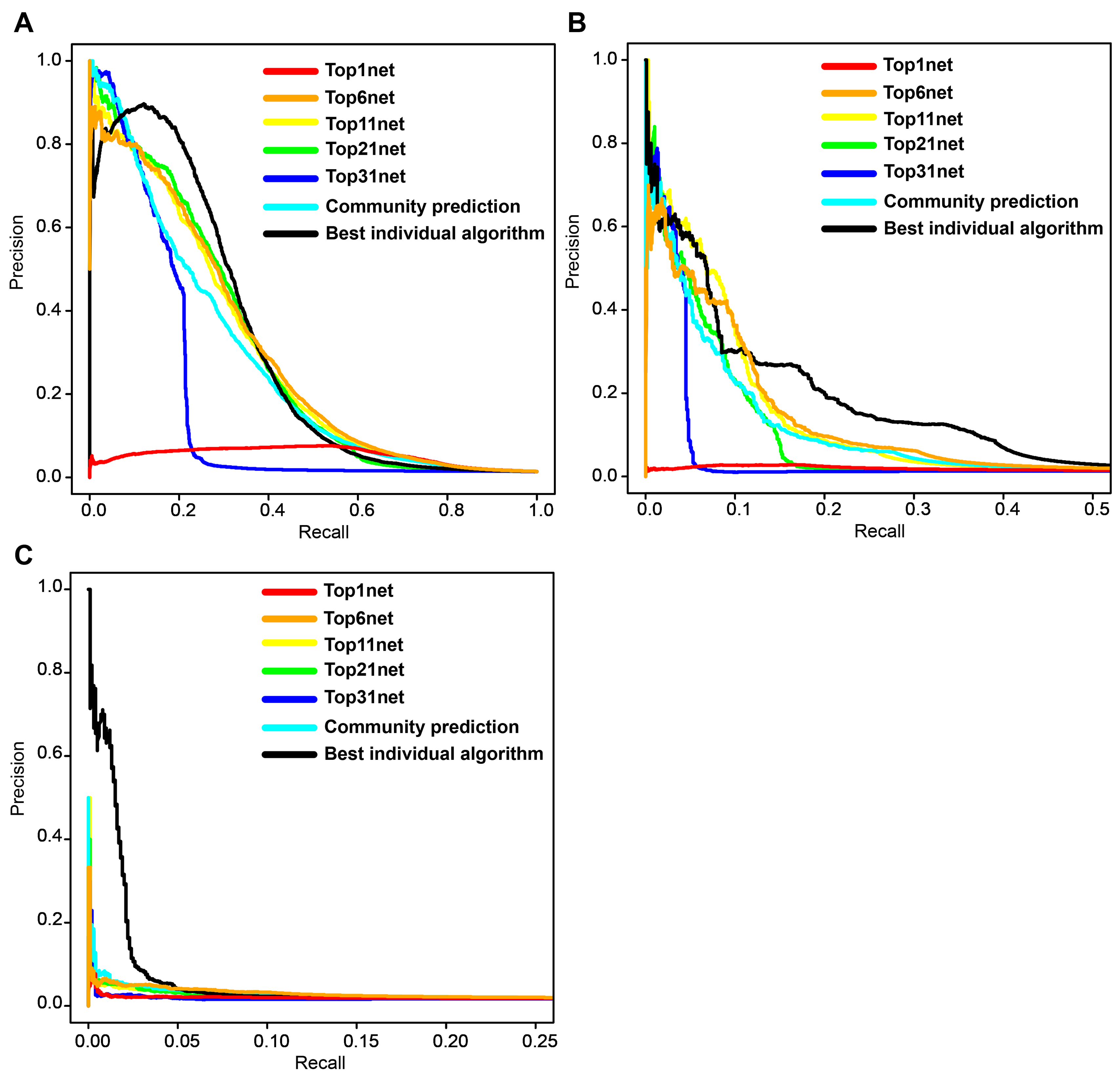

Supplement: Figure S2 — PR curves of Top k Net and community prediction based on integration of the 38 individual algorithms. (A) PR curves for in silico datasets. (B) PR curves for E. coli dataset. (C) PR curves for S. cerevisiae dataset. Vertical and horizontal axes represent precision and recall, respectively. (TIF) [file pcbi.1003361.s002.tif]

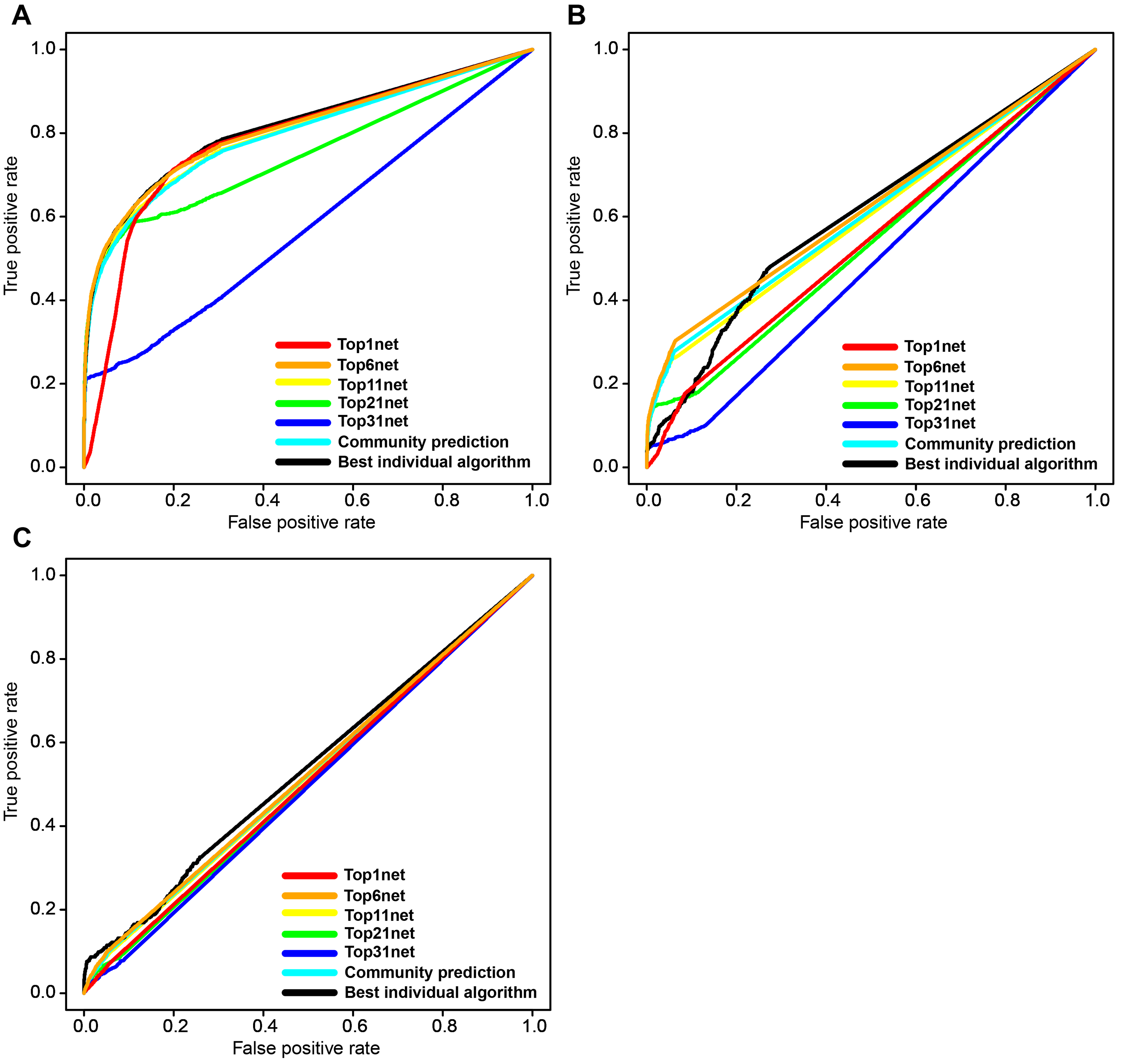

Supplement: Figure S3 — ROC curves of Top k Net and community prediction based on integration of the 38 individual algorithms. (A) ROC curves for in silico datasets. (B) ROC curves for E. coli dataset. (C) ROC curves for S. cerevisiae dataset. Vertical and horizontal axes represent true-positive and false-positive rate, respectively. (TIF) [file pcbi.1003361.s003.tif]

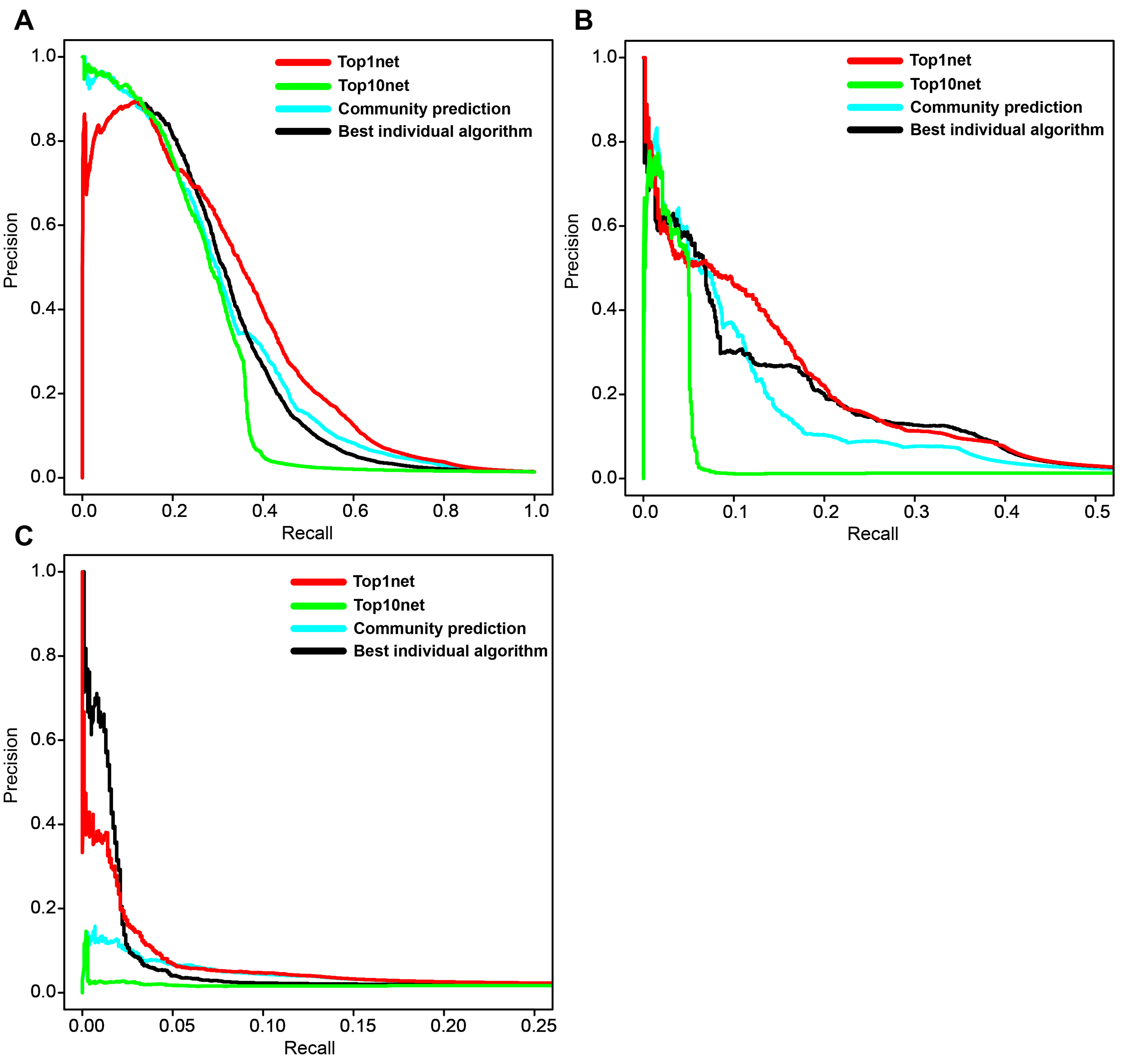

Supplement: Figure S4 — PR curves of Top k Net and community prediction based on integration of the top 10 highest-performance algorithms. (A) PR curves for in silico datasets. (B) PR curves for E. coli dataset. (C) PR curves for S. cerevisiae dataset. Vertical and horizontal axes represent precision and recall, respectively. (TIF) [file pcbi.1003361.s004.tif]

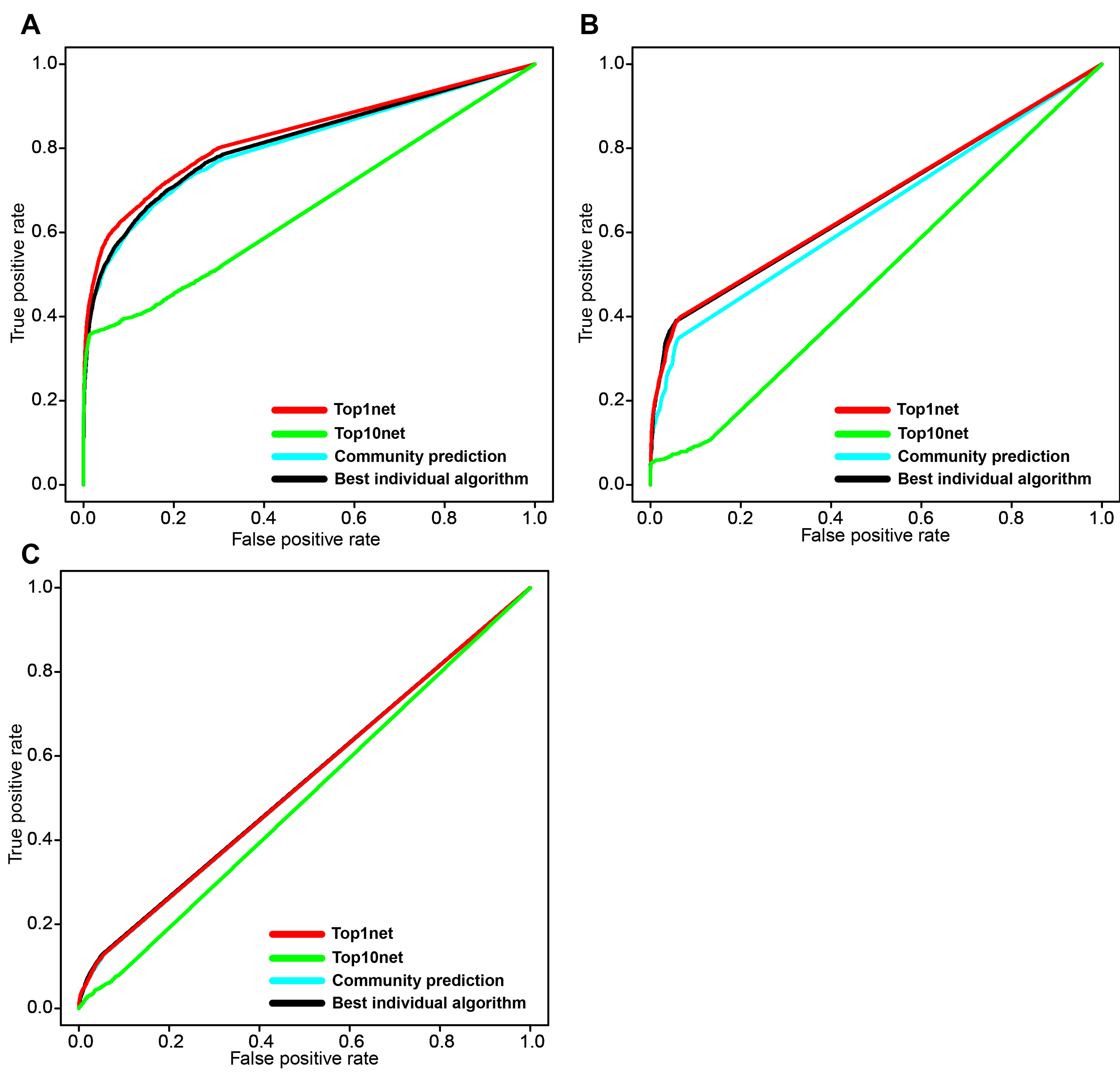

Supplement: Figure S5 — ROC curves of Top k Net and community prediction based on integration of the top 10 highest-performance algorithms. (A) ROC curves for in silico datasets. (B) ROC curves for E. coli dataset. (C) ROC curves for S. cerevisiae dataset. Vertical and horizontal axes represent true-positive and false-positive rate, respectively. (TIF) [file pcbi.1003361.s005.tif]

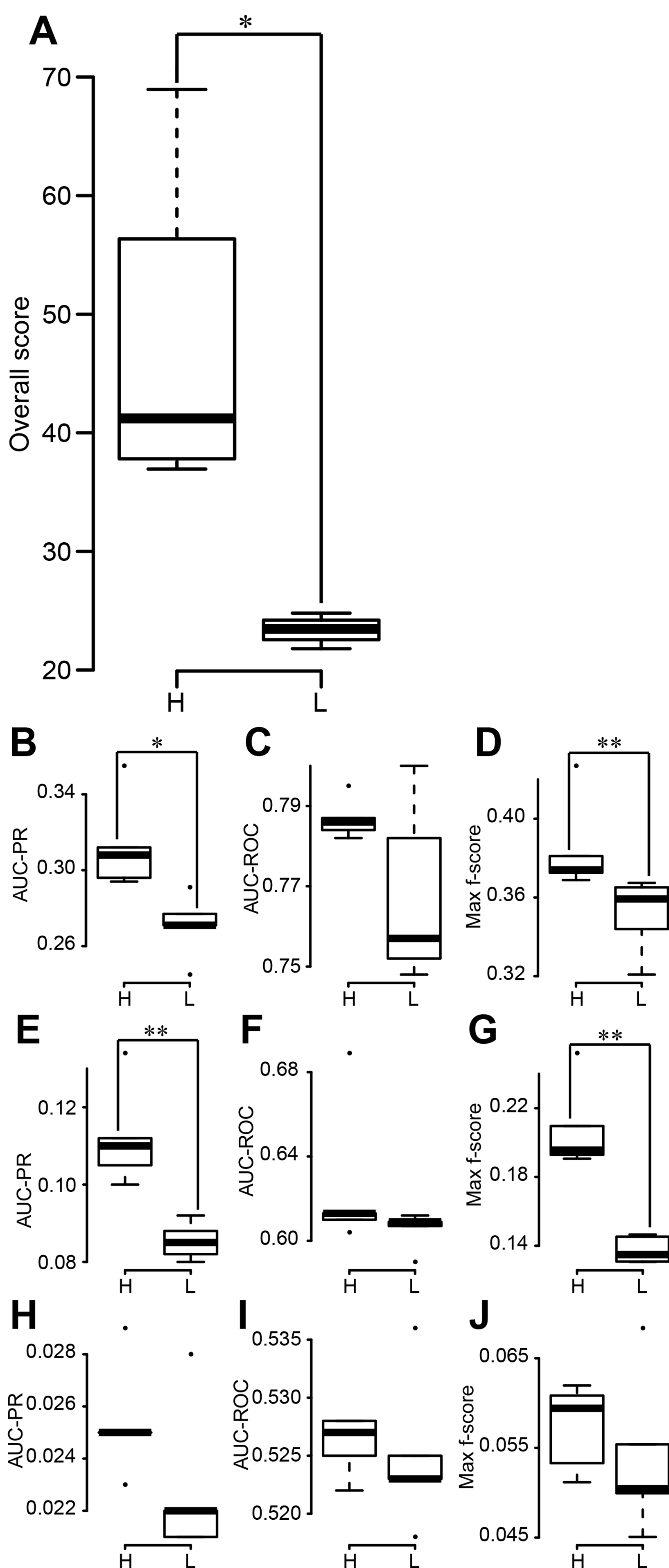

Supplement: Figure S6 — Performances of community prediction based on integration of high- or low-diversity algorithm pairs by EUC distance. H and L represent high-diversity and low-diversity algorithm pairs, respectively. (A) Box-plots of overall score. (B) Box-plots of AUC-PR for in silico dataset. (C) Box-plots of AUC-ROC for in silico dataset. (D) Box-plots of Max f-score for in silico dataset. (E) Box-plots of AUC-PR for E. coli dataset. (F) Box-plots of AUC-ROC for E. coli dataset. (G) Box-plots of Max f-score for E. coli dataset. (H) Box-plots of AUC-PR for S. cerevisiae dataset. (I) Box-plots of AUC-ROC for S. cerevisiae dataset. (J) Box-plots of max f-score for S. cerevisiae dataset. * and ** represent P<0.05 and P<0.01, by the Wilcoxon rank sum test. (TIF) [file pcbi.1003361.s006.tif]

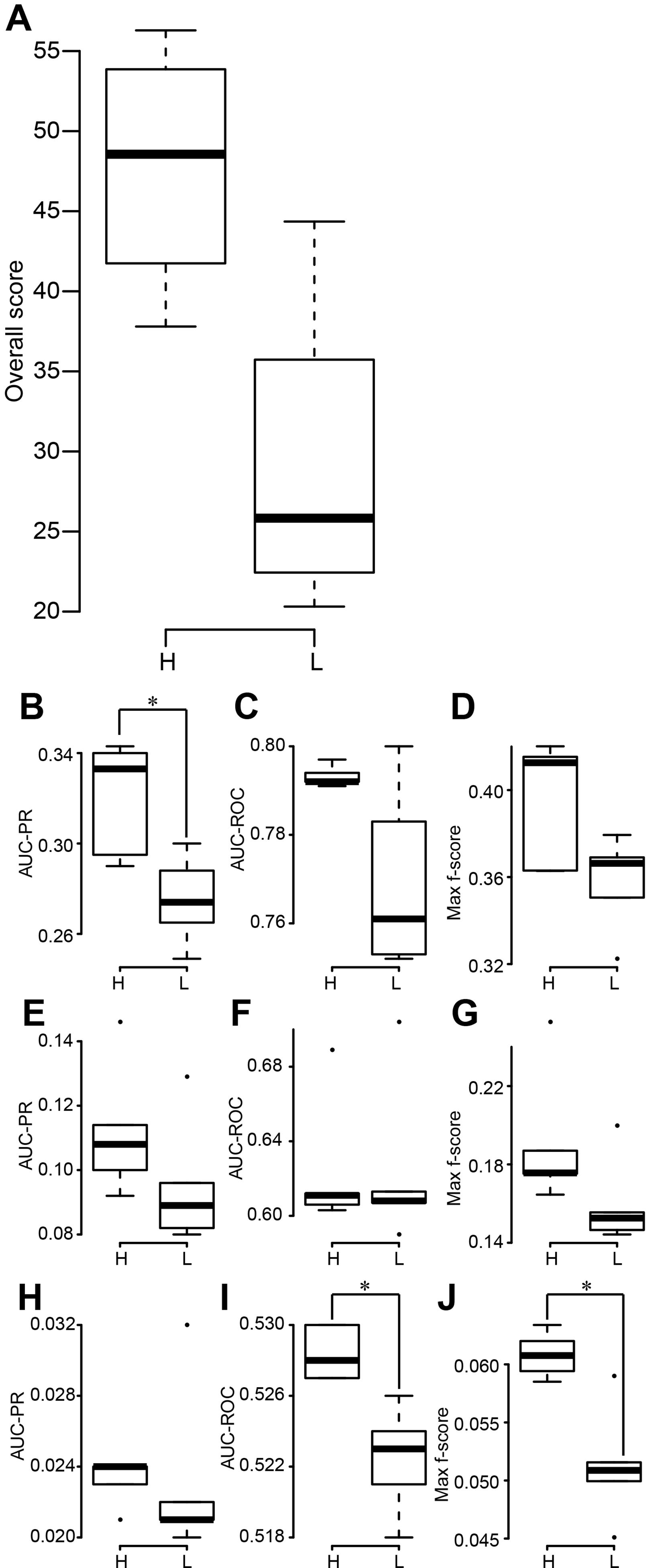

Supplement: Figure S7 — Performances of Top1Net based on integration of high- or low- diversity algorithm pairs by PCA distance. H and L represent high-diversity and low-diversity algorithm pairs, respectively. (A) Box-plots of overall score. (B) Box-plots of AUC-PR for in silico dataset. (C) Box-plots of AUC-ROC for in silico dataset. (D) Box-plots of Max f-score for in silico dataset. (E) Box-plots of AUC-PR for E. coli dataset. (F) Box-plots of AUC-ROC for E. coli dataset. (G) Box-plots of Max f-score for E. coli dataset. (H) Box-plots of AUC-PR for S. cerevisiae dataset. (I) Box-plots of AUC-ROC for S. cerevisiae dataset. (J) Box-plots of max f-score for S. cerevisiae dataset. * represents P<0.05 by the Wilcoxon rank sum test. (TIF) [file pcbi.1003361.s007.tif]

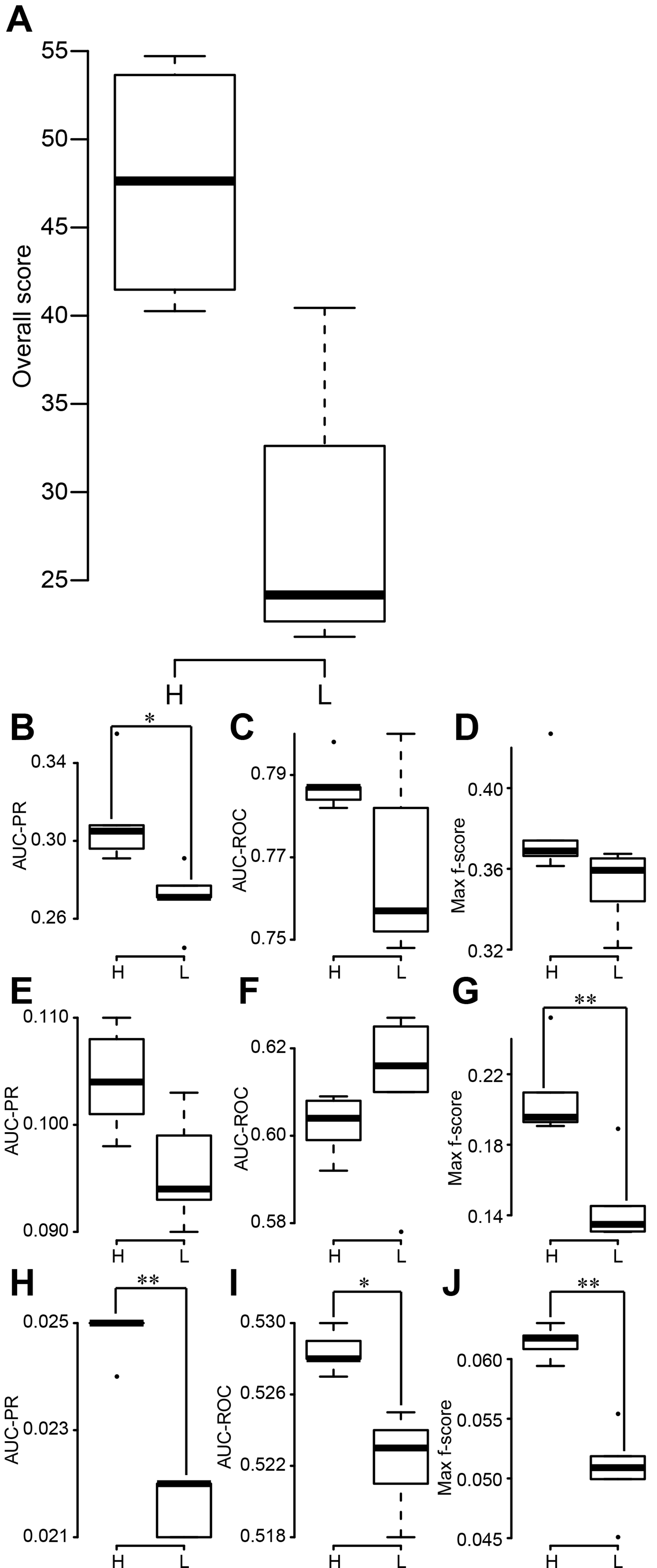

Supplement: Figure S8 — Performances of community prediction based on integration of high- or low- diversity algorithm pairs by PCA distance. H and L represent high-diversity and low-diversity algorithm pairs, respectively. (A) Box-plots of overall score. (B) Box-plots of AUC-PR for in silico dataset. (C) Box-plots of AUC-ROC for in silico dataset. (D) Box-plots of Max f-score for in silico dataset. (E) Box-plots of AUC-PR for E. coli dataset. (F) Box-plots of AUC-ROC for E. coli dataset. (G) Box-plots of Max f-score for E. coli dataset. (H) Box-plots of AUC-PR for S. cerevisiae dataset. (I) Box-plots of AUC-ROC for S. cerevisiae dataset. (J) Box-plots of max f-score for S. cerevisiae dataset. * and ** represent P<0.05 and P<0.01, by the Wilcoxon rank sum test. (TIF) [file pcbi.1003361.s008.tif]

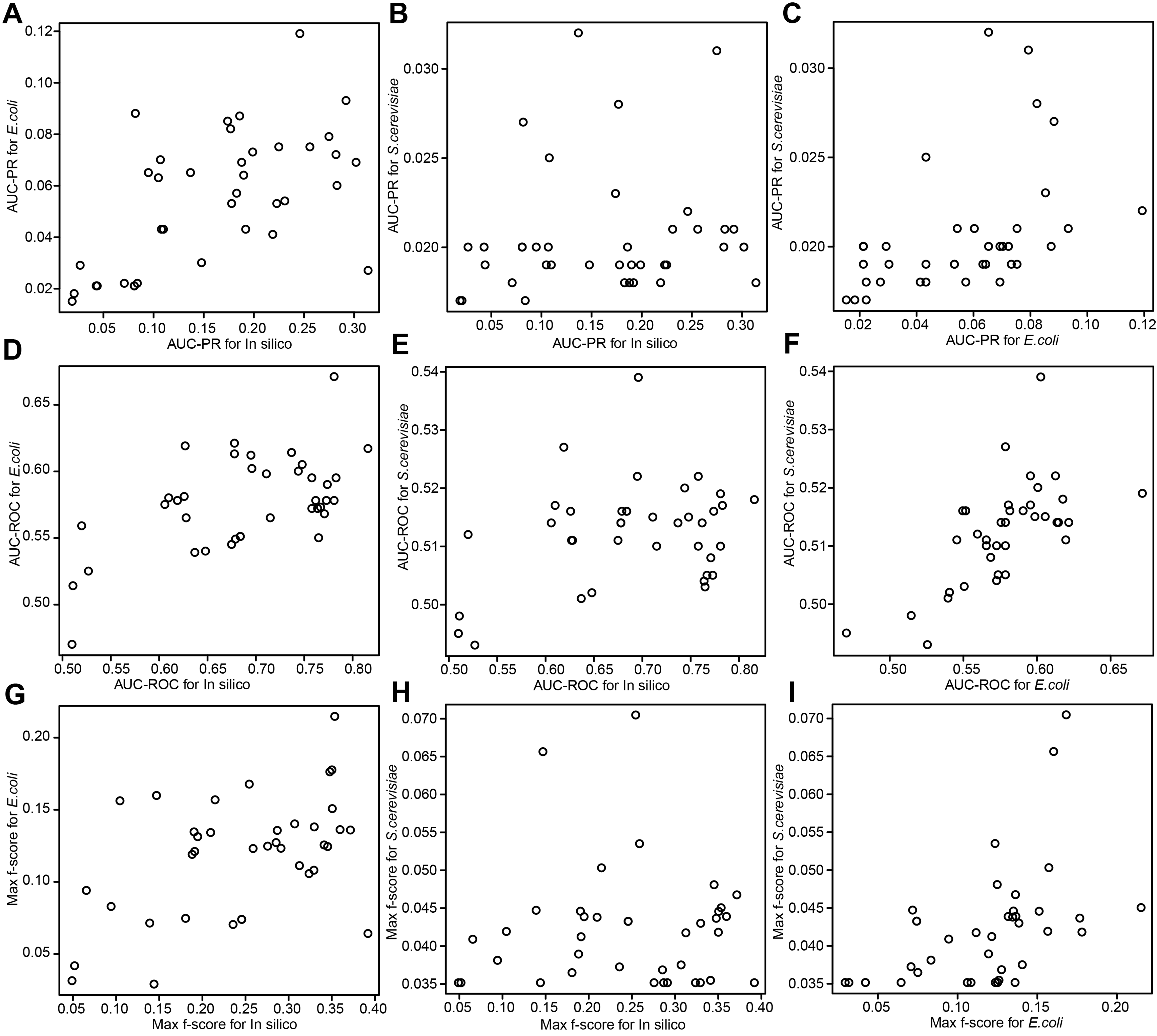

Supplement: Figure S9 — Comparison of algorithm performances across gene-expression datasets. The scatter plots show correlation of algorithm performance between two gene-expression datasets. Vertical axis represents algorithm performance for one gene-expression dataset, while horizontal axis represents that for the other gene-expression dataset. (A) Scatter plots of AUC-PR for in silico and E. coli datasets. (B) Scatter plots of AUC-PR for in silico and S. cerevisiae datasets. (C) Scatter plots of AUC-PR for E. coli and S. cerevisiae datasets. (D) Scatter plots of AUC-ROC for in silico and E. coli datasets. (E) Scatter plots of AUC-ROC for in silico and S. cerevisiae datasets. (F) Scatter plots of AUC-ROC for E. coli dataset and S. cerevisiae datasets. (G) Scatter plots of max f-score for in silico and E. coli datasets. (H) Scatter plots of max f-score for in silico and S. cerevisiae datasets. (I) Scatter plots of max f-score for E. coli and S. cerevisiae datasets. (TIF) [file pcbi.1003361.s009.tif]
