# Supplementary material for: Harnessing Diversity towards the Reconstructing of Large Scale Gene Regulatory Networks
Source: PLoS Comput Biol. 2013 Nov 21;9(11):e1003361. doi: 10.1371/journal.pcbi.1003361 (PMC3836705; doi:10.1371/journal.pcbi.1003361)
Supplement: Table S2 — Correlation coefficient of performance metrics across the DREAM5 gene-expression datasets. The table shows Spearman's correlation coefficient of performance metrics across the DREAM5 gene expression datasets. (DOC) [file pcbi.1003361.s011.doc]

**Supplementary table S**2. Correlation coefficient of performance metrics across the DREAM5 gene-expression datasets.

| **Dataset 1** | **Dataset 2** | **AUC-PR^1^** | **AUC-ROC^2^** | **Max f-score^3^** |
| --- | --- | --- | --- | --- |
| **In silico^4^** | ***E.coli*^5^** | 0.57 | 0.4 | 0.44 |
| **In silico** | ***S.cerevisiae*^6^** | 0.21 | 0.2 | 0.15 |
| ***E.coli*** | ***S.cerevisiae*** | 0.59 | 0.64 | 0.56 |

**^1^**Spearman’s correlation coefficient of AUC-PR between Dataset 1 and Dataset 2. **^2^**Spearman’s correlation coefficient of AUC-ROC between Dataset 1 and Dataset 2. **^3^**Spearman’s correlation coefficient of max f-score between Dataset 1 and Dataset 2. **^4^**In silico Dream 5 dataset. **^5^**Dream 5 dataset from *E.coli*. ^6^Dream5 dataset from *S.cerevisiae*.
